# Is energy excess the initial trigger of carbon overflow metabolism? Transcriptional network response of carbon-limited *Escherichia coli* to transient carbon excess

**DOI:** 10.1186/s12934-022-01787-4

**Published:** 2022-04-21

**Authors:** Zhaopeng Li, Markus Nees, Katja Bettenbrock, Ursula Rinas

**Affiliations:** 1 Helmholtz Centre for Infection Research, Inhoffenstraße 7, 38124 Brunswick, Germany; 2 Technical Chemistry - Life Science, Leibniz University of Hannover, Callinstr. 5, 30167 Hannover, Germany; 3 Max Planck Institute for Dynamics of Complex Technical Systems, Sandtorstrasse 1, 39106 Magdeburg, Germany

**Keywords:** Acetate formation, Acid resistance, Bacterial Crabtree effect, Carbon metabolism, Carbon overfeeding, *Escherichia coli*, Energetics, GABA shunt, Motility

## Abstract

**Background:**

*Escherichia coli* adapted to carbon-limiting conditions is generally geared for energy-efficient carbon utilization. This includes also the efficient utilization of glucose, which serves as a source for cellular building blocks as well as energy. Thus, catabolic and anabolic functions are balanced under these conditions to minimize wasteful carbon utilization. Exposure to glucose excess interferes with the fine-tuned coupling of anabolism and catabolism leading to the so-called carbon overflow metabolism noticeable through acetate formation and eventually growth inhibition.

**Results:**

Cellular adaptations towards sudden but timely limited carbon excess conditions were analyzed by exposing slow-growing cells in steady state glucose-limited continuous culture to a single glucose pulse. Concentrations of metabolites as well as time-dependent transcriptome alterations were analyzed and a transcriptional network analysis performed to determine the most relevant transcription and sigma factor combinations which govern these adaptations. Down-regulation of genes related to carbon catabolism is observed mainly at the level of substrate uptake and downstream of pyruvate and not in between in the glycolytic pathway. It is mainly accomplished through the reduced activity of CRP-cAMP and through an increased influence of phosphorylated ArcA. The initiated transcriptomic change is directed towards down-regulation of genes, which contribute to active movement, carbon uptake and catabolic carbon processing, in particular to down-regulation of genes which contribute to efficient energy generation. Long-term changes persisting after glucose depletion and consumption of acetete encompassed reduced expression of genes related to active cell movement and enhanced expression of genes related to acid resistance, in particular acid resistance system 2 (GABA shunt) which can be also considered as an inefficient bypass of the TCA cycle.

**Conclusions:**

Our analysis revealed that the major part of the trancriptomic response towards the glucose pulse is not directed towards enhanced cell proliferation but towards protection against excessive intracellular accumulation of potentially harmful concentration of metabolites including among others energy rich compounds such as ATP. Thus, resources are mainly utilized to cope with “overfeeding” and not for growth including long-lasting changes which may compromise the cells future ability to perform optimally under carbon-limiting conditions (reduced motility and ineffective substrate utilization).

**Supplementary Information:**

The online version contains supplementary material available at 10.1186/s12934-022-01787-4.

## Background

Many cells, when forced to high metabolic rates under conditions of ample carbon availability, exhibit incomplete carbon catabolism. The phenomenon, characterized by the excretion of metabolic by-products that could otherwise be used for catabolic or anabolic purposes, is generally known as overflow metabolism. Overflow metabolism is a ubiquitous phenomenon found from bacteria to human cells. One example includes the bacterium *E. coli* which changes from fully respiratory metabolism to partly aerobic fermentation and partly respiratory metabolism when carbon availability exceeds energy-efficient coupling of catabolic and anabolic carbon processing. The metabolic change is connected to the excretion of acetate [1].

Overflow metabolism is particularly puzzling as aerobic fermentation is less energy efficient than respiratory metabolism. Although studied for decades the criteria which determine the change from fully respiratory metabolism to partly aerobic fermentation are still unclear. Early hypotheses referred to limitations in the respiratory chain proposing that by-product formation can lead to extra ATP production and to faster growth as long as by-products are formed with a net energy gain [2]. With the objective of maximized ATP synthesis it was later suggested that acetate formation will occur when flux constraints exist either at the level of the NADH turnover rate or the activity of a key TCA cycle enzyme [3].

Since then, various other hypotheses and models have been proposed which are able to predict carbon overflow metabolism, for example high energetic costs for synthesizing the enzymatic inventory for respiratory energy generation [4–7], a limited membrane capacity to harbor respiratory proteins [8], constraints due to cytoplasmic macromolecular crowding [9] and combinations thereof [10]. An analysis of current models revealed that all are able to predict overflow metabolism assuming that cells are in steady state and follow the maximize growth rate principle and implementing two model-specific constraints [11]. Thus, the exact nature of these two biological constraints is still a matter of debate. Moreover, transition states are not implemented in these models and there is no proof that maximum growth rate is the best objective function for cells with the potential for overflow metabolism.

Overflow metabolism is observed in *E. coli* when cells are grown aerobically at conditions of unrestricted carbon supply, namely during exponential growth in batch cultures but also at steady state in balanced continuous culture systems, for example during carbon-limited growth at high(er) dilution rates [12, 13] as well as during N-limited growth at low(er) dilution rates [14]. Moreover, overflow metabolism can occur when cells are exposed to stressful conditions, e.g. during induction of recombinant protein production [15, 16] and also when balanced growing cells in carbon-limited conditions are subjected to carbon excess such as a glucose pulse or a dilution rate upshift [17–20]. Thus, overflow metabolism is observed in balanced as well as unbalanced growth conditions and appears to be the result of restricted anabolic capacities at conditions of ample or surplus carbon supply.

In this study we analyze the response of *E. coli* to a single glucose-pulse in glucose-limited continuous cultures with special attention to the reorganization of the transcriptome and the identification of those transcription and sigma factors which are responsible for organizing the transcriptome change. Our analysis includes the time-dependent transcriptomic reorganization during the period of glucose excess and acetate accumulation as well as the following phases of glucose and acetate depletion and apparent return to the pre-glucose pulse starting point. Thus, we will consider the shift from fully respiratory metabolism to partly aerobic fermentation and back again to full respiratory metabolism. This way we will cover reversible as well as potentially irreversible responses towards a transient exposure to carbon excess conditions.

## Results

**Fig. 1:**
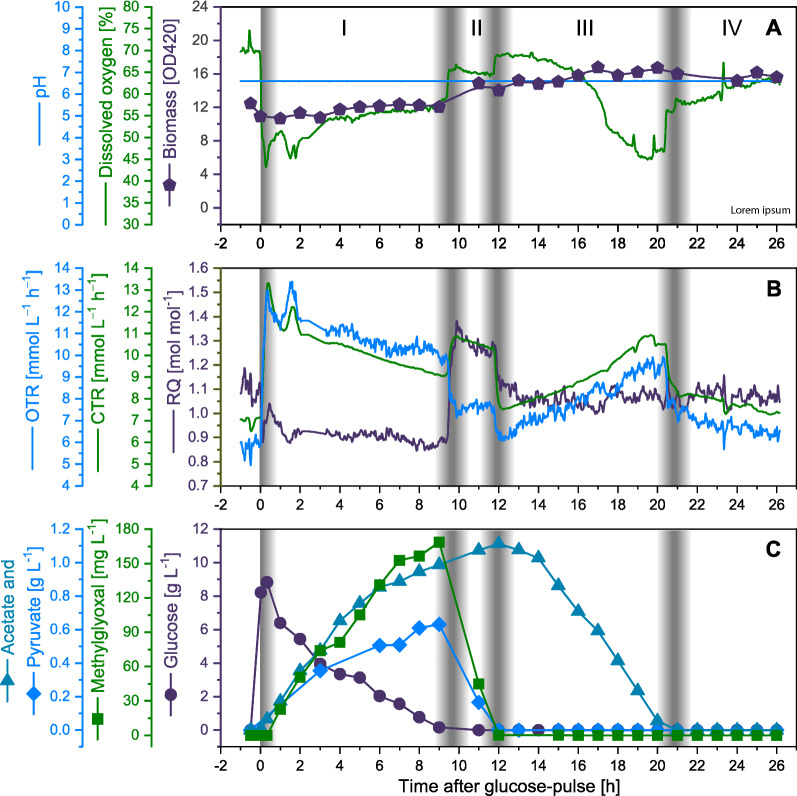
Metabolic response to the addition of a single glucose pulse to a glucose-limited continuous culture. **A** Time course data of biomass and dissolved oxygen concentrations, pH, and **B** volumetric oxygen (OTR) and carbon dioxide transfer rates (CTR) and respiratory quotient (RQ). **C** Time course data of the concentrations of glucose, acetate, pyruvate and methylglyoxal in the cell free culture broth. Time zero indicates the time point of the glucose pulse and the dashed gray shaded areas separate the four distinct metabolic phases after the glucose pulse

Slow-growing cells at steady state in a carbon-limited continuous culture (D = μ = 0.072 h^−1^, biomass concentration approx. 4 g L^−1^) were exposed to sudden carbon excess by a single glucose pulse leading to an increase of the glucose concentration from ~ 0 to 10 g L^−1^. The pH was kept at pH 6.6 and the dissolved oxygen concentration (pO_2_) was always above 40% air saturation revealing constant pH and aerobic conditions during the entire process time, respectively (Fig. 1).

### The macroscopically observable metabolic response of slow-growing cells to sudden glucose excess

The macroscopically observable metabolic response towards the glucose pulse can be roughly classified into four distinct phases which are easily detectable by following the cellular respiratory activity, glucose uptake, and formation and uptake of metabolic by-products (Fig. 1). The initial part of the first phase is characterized by an instantaneous respiratory burst followed by a second peak in respiratory activity and a consecutive slow decrease until the beginning of the second phase. During this first phase encompassing approx. 9 h, cells utilize the excessive glucose concomitant to the formation of organic acids (e.g., accumulation of pyruvate and acetate) and methylglyoxal. Within this phase the respiratory quotient (RQ) dropped from initially 1.1 to 0.9 mol mol^−1^ indicative of incomplete glucose catabolism and formation of organic acids (including also d- and l-lactate and formate, data not shown). The second phase of approx. 2–3 h started after the exhaustion of excessive glucose and is characterized by degradation of pyruvate and methylglyoxal concomitant to a strong increase in the RQ in accordance with the (co)utilization of substrate(s) having a higher degree of reduction than glucose. The beginning of the third phase lasting approx. 8–9 h is manifested by the depletion of pyruvate and methylglyoxal, the onset of acetate degradation and the return of the RQ to pre-glucose pulse values indicating the utilization of substrates with a similar degree of reduction than glucose. The end of the third and beginning of the fourth phase is reached after acetate depletion. Thereafter, cells continue to approach again the (pseudo) steady state connected to the dilution rate of D = μ = 0.072 h^−1^. Biomass increase after the glucose pulse is marginal in particular during the first phase with the consumption of excessive glucose concomitant to the formation of organic acids.

### Which biological processes are affected by transcriptome changes in response to sudden carbon excess?

**Fig. 2:**
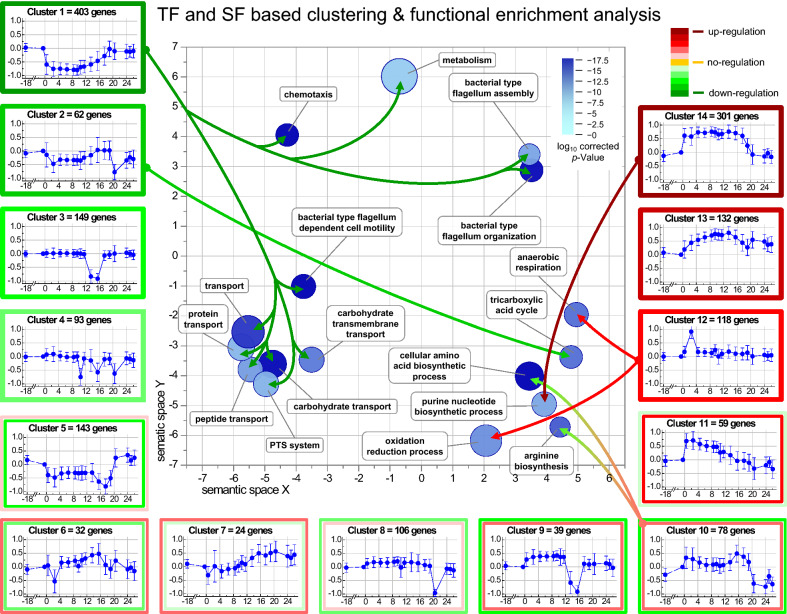
Transcriptome response to sudden carbon excess: determination of affected biological processes. Transcript data of differentially expressed genes with a similar change in the time-course pattern after the addition of the single glucose pulse were partitioned into clusters based on the background information of transcription factor (TF)/sigma factor (SF) − target gene interaction from RegulonDB database (http://regulondb.ccg.unam.mx/) [21, 22]. The constrained k-means clustering charts (Clusters 1–14) show the mean scaled time-dependent gene expression profiles (log_2_ fold change) with standard deviation averaged over the genes belonging to the respective cluster. The number of genes within a specific cluster is indicated on top of the cluster graph. The abscissa shows the time after the glucose pulse in hours and the ordinate the normalized log2 ratio of the differentially expressed genes (with the highest or lowest log2 ratio in the corresponding cluster). Clusters were grouped as follows: down-regulated (Clusters 1–4), non-uniform (Clusters 5–11), and up-regulated genes (Clusters 11–14). Frame color codes indicate down- (green), non-uniform (greenish and reddish) and up-regulation (red) of the genes in the corresponding cluster (see insert for color code meaning, inner and outer frames correspond to non-uniform regulation in the initial or later phases after the glucose pulse, respectively). The semantic map of over-represented GO categories of the ontology “biological process” in the obtained clusters was calculated using the web service “REVIGO” [23] applying the following criteria; number of differentially-expressed genes in the corresponding GO category (represented as disc) belonging to one cluster is larger than 9 and the corrected *p*-value lower than 0.0025 (for details refer to Materials and methods and Additional file 2). The disc color in the semantic map indicates the significance of over-representation of the category (log10 nominal *p*-value, dark blue highly significant, see insert for color code meaning) and the disc size the proportionality to the log number of genes in the category. Clusters containing genes of over-represented GO categories are connected by arrows with the respective GO category discs

To better understand the impact of the sudden carbon excess to cells accustomed to limited carbon availability, the time-dependent change of the transcriptome was analyzed after glucose addition to slow-growing cells in steady state glucose-limited continuous culture. For this purpose, the time-dependent transcript data were clustered based on information of the combined transcription factor (TF)/sigma factor (SF)—target gene interactions as described in the Section “Transcriptome analysis” (Fig. 2, for more details see also Additional file 2).

The analyses revealed that gene transcripts responding to the glucose pulse, 1739 differentially expressed genes (DEGs) in total (absolute log2 fold change ≥ 1.5, corresponding to 41% of all detectable genes on the microarray, see Additional file 2) and exhibiting comparable time course profiles can be grouped into 14 different clusters (Fig. 2). Four clusters (1–4) containing ~ 40% of all DEGs (707 genes) show down-regulation of enclosed genes in response to the glucose pulse. Seven clusters (5–11) with ~ 28% of all DEGs (481 genes) do not reveal a uniform pattern in response to the glucose pulse and the remaining three clusters (12–14) with ~ 32% of all DEGs (551 genes) show up-regulation of enclosed genes after glucose addition.

A functional enrichment analysis revealed that the first group of clusters containing the down-regulated genes encompasses among others also the genes encoding proteins involved in motility, chemotaxis, carbohydrate transport as well as TCA cycle and the last group of clusters containing the up-regulated genes includes amongst others the genes encoding proteins involved in anaerobic respiration, nucleotide and amino acid metabolism (Fig. 2, for more details of the analysis see also Additional file 2).

### Identification of transcription and sigma factors controlling the transcriptional rearrangement in response to the glucose pulse

Transcription factors either activate or repress transcription through their binding to specific sites and sigma factors are required for the formation of the multi-subunit RNA polymerase holoenzyme which recognizes specific promoters and regulates transcription initiation [24]. Thus, gene expression is controlled at the level of TFs as well as at the level of SFs. And moreover, the activity of the majority of TFs is modified through posttranslational modifications, e.g., through binding to metabolites [25].

**Fig. 3:**
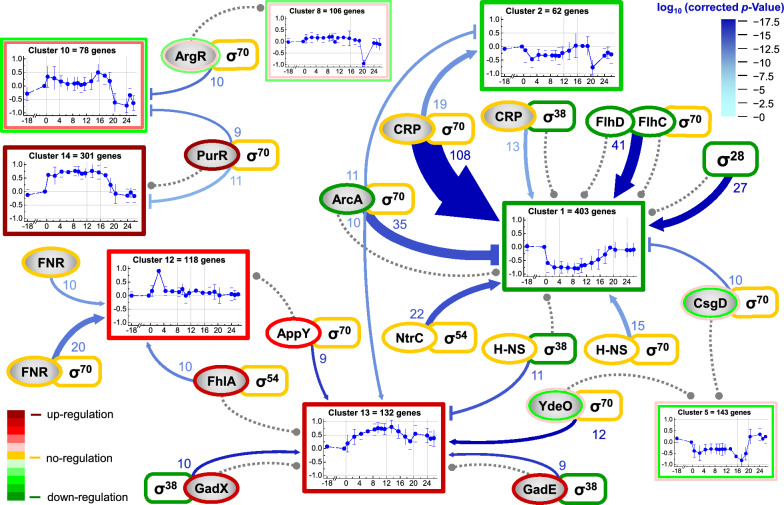
Transcription factor/Sigma factor − target gene regulatory network response to sudden carbon excess. Transcription factor (TF)/Sigma factor/(SF) − target gene network map only considering expressed genes responsive to the addition of the single glucose pulse (absolute log_2_ fold change ≥ 1.5) and TF/SF combinations which have at least significant activating or inhibiting interactions (corresponding corrected *p*-value < 0.0025) with at least 9 genes within one cluster (for details refer to Materials and methods and Additional file 2). Line endings with arrow indicate activation and line endings with bar represent inhibition of genes through respective TF/SF combinations. For example, a line ending with an arrow pointing to a cluster showing down-regulated genes indicates that the respective TF/SF combination loses control. The intensity of the blue color indicates the significance of interaction (log10 nominal *p*-value, dark blue highly significant, see insert for color code meaning). The width of the blue line (and number close to it) connecting TF/SF combination and cluster (clusters as in Fig. 2) indicate the number of genes in the respective cluster controlled by this TF/SF combination. The gray dotted lines connecting TF or SF and cluster indicate the assignment of the TF or SF to a cluster according to its own expression profile. For simplicity, short abbreviations of TFs represent the active form (e.g., CRP represents CRP-cAMP) and a gray background indicates that the active form of the TF is generated by posttranslational modification. The color codes of the frames around the cluster indicate transcriptional down- (green), non-uniform (greenish and reddish) and up-regulation (red) of the genes in the corresponding cluster (see also insert for color code meaning, inner and outer frames correspond to non-uniform regulation in the initial or later phases after the glucose pulse, respectively). The color codes of the frames around the TFs and SFs indicate expression level of respective TF and SF genes, respectively (color code as described above for cluster frames)

For a better understanding of the transcriptional network response towards the sudden carbon excess, a combined TF/SF—target gene network matrix was generated including all known TFs and SFs and the corresponding genes controlled by them (for details refer to Additional file 2). This matrix was used to create a TF/SF—target gene map displaying only those TF and SF combinations which were identified to play a dominant role in the control of the transcriptional response towards the glucose pulse, namely controlling at least 9 genes within one cluster (Fig. 3).

#### Transcriptional reorganisation mainly the result of reduced influence of CRP-cAMP

The TF/SF—target gene map revealed the central metabolic regulator CRP-cAMP [26, 27] in combination with the house-keeping sigma factor RpoD (σ^70^) [28] as the most important TF/SF combination involved in regulating the transcriptional response to the sudden glucose excess. 108 genes activated by the CRP-cAMP/RpoD combination are found in Cluster 1 which contains the majority of all down-regulated genes (403 of the 707 down-regulated genes). The expression of these genes dropped immediately after the glucose pulse, increased slowly after glucose depletion and reached the pre-glucose pulse expression level after complete consumption of the acidic by-products, e.g., acetic acid (Figs. 1 and 3). Thus, CRP-cAMP/RpoD influence on transcription strongly declined after the exposure to excessive glucose and increased again after glucose exhaustion. Another set of 19 genes activated by the CRP-cAMP/RpoD combination are found in Cluster 2 which also contains down-regulated genes but with different time-dependent expression profiles corroborating the loss of influence of the CRP-cAMP/RpoD combination on gene expression after glucose addition (Fig. 3). Moreover, also genes activated by CRP-cAMP and under control of the stationary phase sigma factor RpoS (σ^38^) revealed down-regulation (also found in Cluster 1) pointing to a decreased activity of CRP-cAMP independent of the SF partner.

#### Enhanced influence of ArcA contributes to transcriptome reorganisation

Phosphorylated ArcA [29] in combination with RpoD was identified as another important TF/SF combination involved in regulating central carbon catabolism and participating in the regulation of the transcriptional response to the glucose pulse. Genes repressed by ArcA and transcribed by the RNA(RpoD) polymerase are strongly down-regulated (present in Clusters 1 and 2) and those which are activated by the ArcA/RpoD combination are strongly up-regulated (present in Cluster 13). Thus, the influence of ArcA/RpoD revealed a clear increase, opposite to the decreasing influence of cAMP-CRP, in response to the glucose pulse.

#### Various TFs and SFs contribute to motility reduction

Another set of regulatory factors with strong reduction in their control of gene expression concerns the pair FlhDC/RpoD and RpoF (σ^28^) (also in Cluster 1). FlhD and FlhC are regulatory proteins which are known as positive master regulators controlling the expression of flagella-related genes which encode proteins required for active cell movement [30, 31]. Expression of *flhDC* is regulated by various environmental factors and also under positive control of the central master regulator CRP-cAMP [30, 31]. FlhD and FlhC also regulate the expression of *fliA* encoding RpoF which is an alternative flagella-specific sigma factor that acts on the second level of flagella formation control by targeting RNA polymerase to flagella-related genes [30–32]. Interestingly, carbon-limited continuous culture experiments revealed that flagella abundance is correlated with growth rate, with faster growing cells producing more flagella than slower growing ones [33].

Another set of genes found in Cluster 1 concerns down-regulated genes which are under negative control of CsgD in combination with RpoD. CsgD is a transcriptional activator involved in the activation of genes witch are required for curli production and biofilm formation [34]. Thus, down-regulation of genes under negative control of CsgD indicates activation of biofilm forming genes in line with a general motility reduction as the response to glucose excess.

#### NtrC, N-NS, ArgR and PurR are four more important TFs loosing influence

Further sets of genes found in Cluster 1 concern those activated by NtrC in combination with the Nitrogen-responsive sigma factor RpoN (σ^54^) and the histone-like nucleoid structural protein (H-NS) in combination with RpoD. Loss of H-NS influence on gene expression is also demonstrated by a set of 11 up-regulated genes found in Cluster 13 which are under negative control of the H-NS/RpoS combination (Fig. 3). Other TFs which loose influence through negative control are ArgR and PurR (found in Clusters 10 and 14, respectively), both in combiantion with RpoD. ArgR is a pleiotropic repressor originally discovered as a repressor of arginine biosynthesis when bound to its corepressor l-arginine [35]. PurR is a repressor initially recognized as regulatory factor of purine metabolism but now recognized as a more general transcription factor activated by hypoxanthine [36] the final degradation product of adenine nucleotides such as ATP [15].

#### TFs controlling anaerobic genes and acid resistance are gaining influence

Other TF/SF combinations additionally contributing to the regulation of the cellular response towards sudden glucose excess include TF/SF pairs gaining more influence through positive control (FNR/RpoD, FhlA/RpoF, GadXE/RpoS, YdeO/RpoD, AppY/RpoD, found in Clusters 12 and 13).

FNR can act as an expression repressor or activator depending on the properties of the gene or the presence of other binding partners. Activation of FNR as transcription regulator occurs through iron-sulfur-clusters and is thought to exclusively take place in the absence of oxygen [37, 38]. As the cells did not experience oxygen limiting conditions during the experiment another not yet identified redox active compound may additionally influence the activity of FNR. Or potential oxygen limitation in the cytoplasm may contribute to FNR activation at high respiration rates despite sufficient oxygen in the culture broth. FhlA is a formate-dependent transcriptional activator controlling expression of genes mainly related to hydrogen formation [39–41]. GadX/E, YdeO and AppY are involved in the regulatory control of the acid response, a complex inducible cellular response providing protection against low pH [42–46].

Altogether, these findings suggest that the major and central reaction to carbon excess is geared towards a general reduction of carbon processing capacities including the down-regulation of gene expression connected to carbon uptake and catabolism as well as mobility. This change is mainly transient and cells increase the expression of the majority of these genes again after the consumption of excessive carbon. The master regulator orchestrating this process is clearly CRP-cAMP which also controls the expression of other transcription factors (e.g., *flhDC* but also *gadX/E,* see also https://ecocyc.org/) [47]) involved in the regulation of the transcriptional response towards the glucose upshift.

### Cooperativity and timing of the transcriptional response to the glucose pulse through different TF/SF combinations

Most genes are not controlled through a single TF/SF combination but possess multiple binding sites for different transcription and sigma factors, a fact that introduces eminent complexity to the transcriptional response towards environmental perturbations. For example, *aceE*, the gene coding for one of the three different subunits of the pyruvate dehydrogenase complex, is under positive control of CRP-cAMP and under negative control through phosphorylated ArcA (https://ecocyc.org/) [47]. Negative or positive control of *aceE* through FNR depends on further binding factors, and in addition to CRP-cAMP, ArcA and FNR four other transcription factors and two sigma factors, namely RpoD and RpoS, are involved in controlling *aceE* expression.

**Fig. 4:**
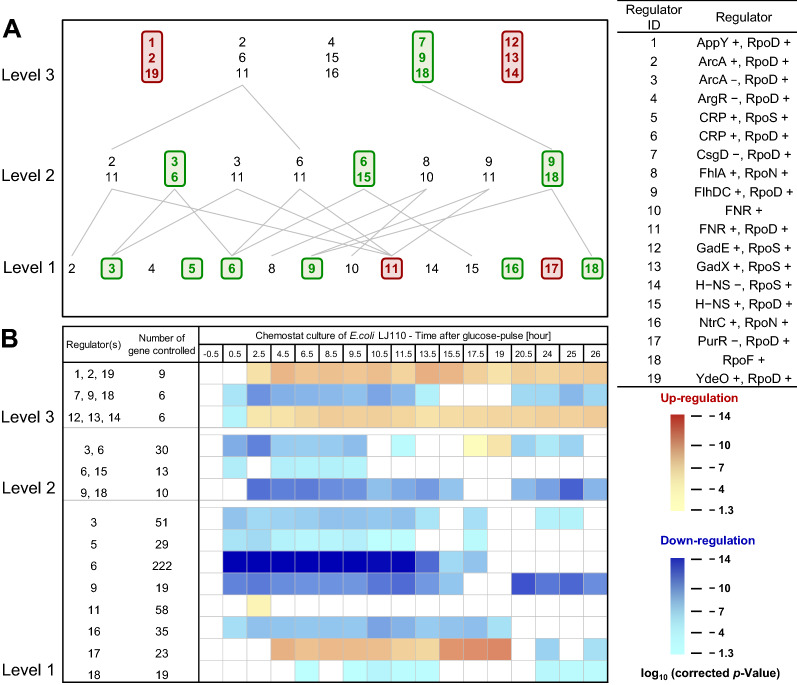
Time-dependent change of the TF/SF co-regulatory network to sudden carbon excess. The extent and time-dependent change of co-regulation of the transcriptional response through TSs and SFs and their different combinations were determined considering only those factors which were identified as important in the independent TF/SF—target gene regulatory network analysis (presented in Fig. 3). **A** Level 1 contains single TF/SF combinations which control gene expression without co-control of the other identified important regulator combinations and, for example, Level 2 includes sets of two TF/SF combinations together controlling gene expression without co-control of the other identified important regulator combinations. A TF/SF combination or a set of TF/SF combinations which are subgroups in the next higher layer are connected to the respective groups with gray lines. A TF/SF combination or sets of TF/SF combinations which have corrected *p*-values lower than 0.05 (Fisher’s exact test and two sample t-test) are considered as decisive and shown with bold letters in colored boxes, green and red corresponds to a TF/SF combination or sets of TF/SF combinations leading to down- or up-regulation of controlled genes, respectively. **B** The significance of regulation of gene expression through a single TF/SF combination or sets of TF/SF combinations at given time points after the glucose pulse is shown as heat-map in comparison to the expression of all other genes using a one-sided two sample t-test. Blue and red colors indicate down- and up-regulation, respectively. Darker colors represent lower corrected *p*-values indicative of more significant regulatory control through the respective single TF/SF combinations or sets of TF/SF combinations at the indicated time points (For more details see Analysis of expression data in section “Materials and methods” and Additional file 2)

To get a more detailed insight into the regulatory control of the transcriptome change we analyzed potential cooperative and time-dependent changes of transcription control through different TF/SF combinations. To render this analysis feasible only those TF/SF combinations were considered which were identified beforehand as important (shown in Fig. 3). These analyses generated a time-dependent heat-map of the activity of TF/SF combinations acting alone or in cooperative control with the other beforehand identified TF/SF combinations (Fig. 4).

#### Loss of CRP-cAMP influence independent of co-control through the other as important identified TFs is largely responsible for the transcriptome change

The analyses identified again CRP-cAMP/RpoD as the most important regulatory unit (Fig. 4). CRP-cAMP/RpoD alone without cooperative control of any of the other as important identified TF/SF combinations was identified as being responsible for the expression change of the largest number of differentially expressed genes (222 genes, Level 1, Fig. 4). Loss of control through CRP-cAMP/RpoD started immediately after the glucose pulse with a strong decrease of the expression of genes under positive control of CRP-cAMP/RpoD. The expression of these genes increased again after depletion of pyruvate and methylglyoxal and reached again the pre-glucose pulse expression level after consumption of acetate (Figs. 1 and 4). A similar time course profile was also found for the expression of genes under positive control of CRP-cAMP and RpoS not cooperatively controlled by any of the other as important identified TF/SF combinations (29 genes, Level 1, Fig. 4) corroborating the loss of CRP-cAMP control independent of the SF partner.

#### Enhanced influence of ArcA modifies the expression time profile of genes co-controlled by CRP-cAMP

Slightly different time-dependent expression profiles were observed for genes under negative control of ArcA/RpoD (51 genes, Level 1, Fig. 4) and under combined negative control through ArcA/RpoD as well as positive control through CRP-cAMP/RpoD (30 genes, Level 2, Fig. 4). The expression of these genes decreased during the period of glucose excess, increased again during the period of pyruvate and acetate consumption, revealed another though transient decrease after acetate depletion and approached thereafter again the pre-glucose pulse expression level.

#### Persistently reduced expression of motility related genes results from co-control through different TFs

A similar profile of reduced expression during the period of glucose excess, transient increase in the period of by-product consumption followed by an even more prominent decrease after depletion of acetate was found for genes involved in motility and chemotaxis (Fig. 4). Genes following these profiles included those under positive control of FlhDC/RpoD (19 genes, Level 1, Fig. 4) or RpoF (19 genes, Level 1, Fig. 4), under combined positive control of FlhDC/RpoD and RpoF (10 genes, Level 2, Fig. 4) and those under combined positive control of FlhDC/RpoD and RpoF and negative control of CsgD/RpoD (6 genes, Level 3, Fig. 4). The expression of these genes did not return to their original pre-glucose pulse levels within the time period investigated.

#### Loss of CRP-cAMP/RpoD or NtrC/RpoN influence on expression of genes not co-controlled by any of the other identified TF/SF combinations reveals similar time profiles

Another set of genes displaying reduced expression after the glucose pulse and having a similar time profile than those under positive control of CRP-cAMP/RpoD included genes under positive control of NtrC/RpoN (35 genes, Level 1, Fig. 4). These so-called nitrogen-responsive genes revealed reduced expression during the presence of glucose and also during the period of acetate consumption. Their function is related to a great diversity of different tasks including transport (e.g., glutamine, dipeptides) and degradation (e.g., arginine, pyrimidine). To many of them no clear functions are yet assigned. For example, for *yeaG* it is only known that expression is increased during stationary phase and acid and salt stress.

#### H-NS influence reduces the time period of reduced expression of genes co-controlled by CRP-cAMP

The final group of genes displaying reduced expression after the glucose pulse included genes under positve control of CRP-cAMP/RpoD as well as H-NS/RpoD (13 genes, Level 2, Fig. 4). The expression of these genes also decreased immediately after glucose addition but already returned to pre-glucose shift levels after glucose depletion (Fig. 4). Among them are the genes encoding FlhD and FlhC, the transcription factors involved in the regulation of flagella-related genes.

#### RpoF control delays decline of gene expression

The majority of genes exhibiting lower expression after the glucose pulse revealed an immediate expression decline with the exception of those under control of RpoF and combined control of FlhDC/RpoD (10 genes, Level 2, Fig. 4) and RpoF (18 genes, Level 1, Fig. 4) which displayed a delayed onset of expression decline after glucose addition.

#### Genes with enhanced expression reveal delayed expression change, particularily those under negative control of PurR and not co-controlled by any of the other identified TFs

The genes with enhanced expression after the glucose pulse revealed an even longer delay before expression changes became detectable (Fig. 4). This is most obvious for genes under negative control through PurR/RpoD (23 genes, Level 1, Fig. 4). These genes are not co-controlled by any of the other as important identified TF/SF combinations. Derepression did not become evident before 3–4 h after glucose addition and their pre-glucose pulse expression level was reached again after complete consumption of glucose and by-products including acetate (Figs. 1 and 4).

#### Genes under control of FNR and not co-controlled by any of the other identified TFs reveal the shortest period of enhanced expression

The shortest time frame of enhanced expression was observed for genes under positive control of FNR/RpoD and not co-controlled by any other important TF/SF pair (58 genes, Level 1, Fig. 4). The expression of this rather big group of genes increased with delay after glucose addition and returned to pre-glucose pulse expression levels quickly after the initial respiratory burst phases well before depletion of glucose. Presumably, expression of these genes reflects cytoplasmic anoxia during enhanced respiratory metabolism independent of sufficient oxygen supply in the culture medium or the accumulation of a yet not identified redox active compound.

#### Delayed but persistent enhanced expression of acid resistance genes under control of multiple TF/SF combinations

The expression of two other sets of genes also increased with delay after the addition of glucose, but remained high for the entire time of the experiment even after depletion of acetate and the return to the pseudo-steady state of the pre-glucose pulse culture conditions. Both of them are under control of triple TF/SF combinations, namely the triple combination AppY/RpoD, ArcA/RpoD, YdeO/RpoD (9 genes, e.g., *hyaA*, Level 3, Fig. 4) and GadE/RpoS, GadX/RpoS, H-NS/RpoS (6 genes, e.g., *hdeA, mdtE*, Level 3, Fig. 4). The transcription factors AppY, YdeO, GadE and GadX are in control of the acid response, a very complex cellular response connected to resistance against low pH. Genes included in these groups are, e.g., *hyaA*, encoding a subunit of a hydrogenase which oxidizes hydrogen to water, *mdtE*, encoding a membrane fusion protein of a putative tripartite efflux pump complex, and *hdeA*, encoding an energy-independent chaperone that protects periplasmic proteins from acid-induced aggregation. HdeA is presumably a member of the acid resistance system 1 (AR1) [45] and mutations in *hdeA* are connected to extreme acid sensitivity. However, for most acid-inducible genes the cellular function is not yet fully understood.

All genes controlled by the above mentioned TF/SF combinations are found in the Additional file 2, short descriptions of gene functions are accessible in (https://ecocyc.org/) [47].

### Some examples of transcriptional reorganization of pathways and processes

Not all affected biological processes and metabolic pathways might be identifiable by the above regulatory target gene network analysis. In particular, if a certain TF/SF combination controls only few genes which may occupy metabolic key positions or if a TF/SF combination antagonizes the regulatory effect of other TF/SF combinations. Also, genes are not included in the above analysis which do not show any significant change in response to the glucose pulse.

**Fig. 5:**
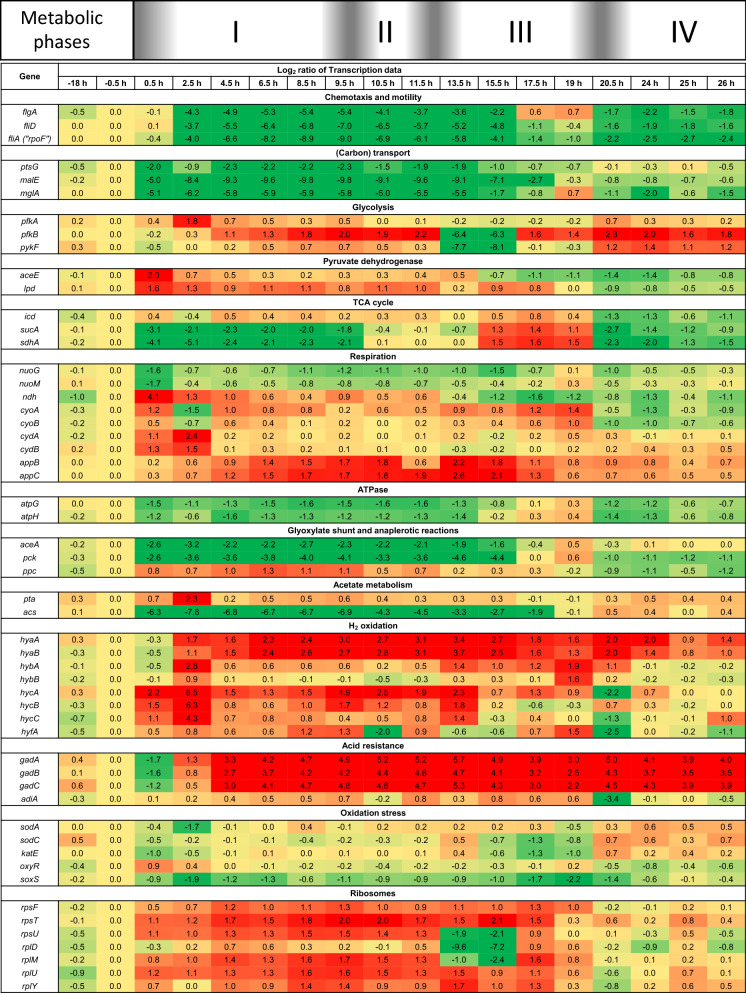
Transcriptional reorganization presented by means of selected metabolic pathways and processes. Heat-map of time course profiles of transcripts (log_2_ fold change) encoding designated proteins involved in chemotaxis, glucose uptake, central carbon and energy metabolism, stress responses and anabolic processes. Time zero indicates the time point of the glucose pulse. Gene names are given to the left. The four distinct metabolic phases indicated on top of the heat-map are as specified in Fig. 1. Box colors indicate transcriptional down- (green), no (yellow) and up-regulation (red) of genes after glucose addition. Box numbers correspond to log_2_ fold transcript changes. More details including extended datasets and pathway maps are found in Additional files 1 and 2

Thus, a selected evaluation of some processes and pathways and their corresponding genes including motility and chemotaxis as well as carbon catabolism including sugar uptake, glycolysis, TCA cycle, respiration, anaplerotic pathways and by-product formation will be given (Fig. 5). Moreover, the analysis will also address some stress responses namely the so-called acid response and oxidative stress response as well as some explicit anabolic processes including biogenesis of ribosomes (Fig. 5).

#### Reorganizing motility and chemotaxis

As already identified by the above described regulatory target gene network analysis, expression of genes facilitating active movement are down-regulated in response to the glucose pulse. This is exemplified by the down-regulation of genes involved in flagella formation (e.g., *flgA*, *fliD*) and also in the down-regulation of the minor flagella-related SF RpoF (“*fliA*”). Down-regulation of these genes started with delay but persisted even after depletion of acetate.

#### Reorganizing carbon catabolism

The functional enrichment analysis already revealed a general down-regulation of catabolic pathways after the glucose pulse (Fig. 2). This analysis is extended now by more detailed studies on changes of gene expression related to carbon catabolism.

##### Sugar transport

Down-regulation of genes involved in sugar transport started immediately after the glucose pulse and increased again with the onset of acetate consumption (e.g., *ptsG*, *malE*, *mglA*). Down-regulation was particularly strong for genes of the maltose transport system (e.g., *malE*). These genes are additionally activated by MalT, a transcriptional activator not identified by the above network analysis as it controls only few genes [48].

##### Glycolysis

Expression of glycolytic pathway genes increased after the glucose pulse, including expression of *pfkA* encoding 6-phosphofructokinase 1, the pacemaker enzyme of glycolysis.

##### Pyruvate dehydrogenase and TCA cycle

The genes encoding the subunits of the pyruvate dehydrogenase (e.g., *aceE*, *lpd*) increased in response to the glucose pulse although these genes are activated by CRP-cAMP/RpoD and repressed by ArcA/RpoD (https://ecocyc.org/) [47]. Thus, the otherwise observed loss of influence of CRP-cAMP/RpoD and increased influence of ArcA/RpoD in response to the addition of glucose is abolished for these genes through interference with other transcriptional regulators. Likely candidates are FNR and PdhR. FNR modifies expression of pyruvate dehydrogenase encoding genes depending on the presence of other transcriptional regulators (https://ecocyc.org/) [47]. An even more likely candidate leading to the enhanced expression of pyruvate dehydrogenase encoding genes is PdhR, the transcriptional regulator of the pyruvate dehydrogenase complex, which loses its repressing influence on controlled genes upon binding to pyruvate [49, 50]. The observed accumulation of pyruvate (Fig. 1) also suggests elevated intracellular pyruvate levels and a corresponding abolished repression of these genes through PdhR, which may alleviate the otherwise strong down-regulation of CRP-cAMP activated and ArcA repressed genes. PdhR was not identified by our network analysis as it controls only few genes and moreover antagonizes expression control though other regulatory factors. Thus, it is advised to additionally check expression data manually for unexpected performance in particular of metabolic key enzymes.

Expression of TCA cycle genes decreased immediately and strongly after glucose addition (e.g., *sucA*, *sdhA*) with the exception of the genes encoding isocitrate dehydrogenase (*icd*) and lipoamide dehydrogenase (*lpd*), the subunit of the pyruvate dehydrogenase as well as the α-ketoglutarate dehydrogenase complex, respectively. The TCA cycle enzyme isocitrate dehydrogenase controls the branch-point at the TCA cycle and glyoxylate shunt and catalyzes the formation of α-ketoglutarate, the substrate of the α-ketoglutarate dehydrogenase. Interestingly, knockout mutants of *lpd* produce more pyruvate and glutamate under aerobic conditions [51].

##### Respiratory energy generation

Expression of genes encoding the subunits of the energy conserving NADH dehydrogenase *nuo* [52, 53] decreased immediately after addition of glucose (Fig. 5). In contrast, expression of *ndh*, the less energy efficient NADH dehydrogenase [54, 55] revealed an opposite time course pattern, increasing immediately after the glucose pulse. The transcripts of the energy efficient terminal oxidase *cyo* [56] revealed a more complex time course pattern with an initial moderate increase followed by a short transient decrease and slight upregulation during the phases of glucose excess and by-product consumption. The transcripts of less energy efficient oxidases (*cyd* and *app*; [57, 58]) increased strongly after glucose addition, however each with a different time course pattern. Expression of *cyd* increased rapidly, peaked at the time when expression of *cyo* strongly decreased (corresponding to the time after the initial respiratory burst phases, 2.5 h after the glucose pulse) and returned quickly thereafter to almost pre-glucose pulse expression levels. Expression of the less energy efficient terminal oxidase *app* increased with delay but maintained a very high expression level during the period of glucose excess and by-product consumption. Transcription of genes encoding ATP synthase subunits (e.g., *atpG*, *atpH*) decreased immediately after glucose addition and their transcript levels remained low during glucose excess and by-product formation.

##### Anaplerotic reactions

Transcription of genes encoding the glyoxylate bypass enzyme isocitrate lyase (*aceA*) and phosphoenolpyruvate (PEP) carboxykinase (*pck*) decreased strongly and instantaneously after the glucose pulse presumably contributing to a reduced capacity to utilize acetate as a substrate. In contrast, expression of the anaplerotic PEP carboxylase (*ppc*), the partner enzyme of the anaplerotic futile cycle pair *pck*/*ppc*, increased after glucose addition. Conversion of PEP to pyruvate via pyruvate kinase leads to ATP formation while PEP carboxylase catalyzed formation of oxaloacetate from PEP does not lead to ATP production.

##### Acetate metabolism

Expression of *pta* increased after glucose addition revealing an almost identical expression profile as the less energy efficient *cyd* terminal oxidase; peaking after the initial respiratory burst phases (2.5 h after the glucose pulse) and returning thereafter to almost pre-glucose pulse expression levels. In contrast, expression of the acetate scavenging *acs* decreased instantaneously and strongly after the glucose pulse maintaining very low expression levels during the time of glucose excess and by-product formation.

##### Hydrogen formation

Interestingly, all genes encoding the different hydrogen forming enzymes (hydrogenases 1–4, *hya*, *hyb*, *hyc*, *hyf*) revealed increased expression after the glucose pulse. In particular, expression of the genes encoding the non-energy conserving hydrogenese 3 (*hyc*) increased immediately after glucose addition with a peak expression 2.5 h after the glucose pulse (corresponding to the time after the initial respiratory burst phases, 2.5 h after the glucose pulse). The genes of the other hydrogen forming enzymes (*hya*, *hyb*) revealed a slower expression increase but maintained a high expression level during a longer time period.

#### Longterm changes—induction of the acid response

Gene expression analysis revealed a set of genes that maintained persistent and strong upregulation long after expression of the majority of other genes returned to their pre-glucose pulse expression levels. These genes are members of the acid resistance system 2 (AR2), the so-called GABA shunt, which is induced at low pH and confers cell resistance to extreme acidic conditions. AR2 is a stationary phase-induced and glutamate-dependent system that requires glutamate decarboxylase (*gadA*, *gadB*) and a glutamate: γ-aminobutyric acid (GABA) antiporter (*gadC*), as well as exogenous glutamate [44, 45]. In AR2 glutamate uptake is coupled to proton export, carbon dioxide formation and excretion of GABA. Apart for the first sampling point (30 min after glucose addition), all three genes, *gadA*, *gadB* and *gadC* exhibited very strong upregulation which persisted during the entire experiment (Fig. 5). In addition, proteome analyses revealed an approx. tenfold increase of GadA which finally reached 0.8% of the cellular proteome (pre-glucose shift level, 0.08% of cell proteome, data not shown). Induction of the arginine dependent acid resistance system (AR3, *adiA* [45]) was also observed, but less intensive and not persistent (Fig. 5). It should be noted that the culture pH did not decrease below pH 6.6 during the entire experiment.

#### Oxidative stress response

It has been hypothesized that overflow metabolism could be connected to oxidative stress and interpreted as an attempt to reduce the generation of reactive oxygen species (ROS) [59]. We thus analyzed the expression of genes involved in the defense against oxidative stress. Our analysis did not reveal any significant induction of genes involved in oxidative stress protection after the glucose pulse (e.g., *sodA*, *sodC*, *katE*, *oxyR*, *soxS*, Fig. 5). On the contrary, expression of *soxS*, a dual transcriptional activator which participates in the removal of superoxide, even declined after glucose addition.

#### Reorganizing anabolism—expression of ribosomal genes

The content of ribosomes increases with increasing growth rate [60], this way reflecting the anabolic capacity of growing cells. After the addition of glucose, expression of genes encoding ribosomal proteins of both small and large subunits revealed a clear increase (Fig. 5). Ribosomal genes are found in different clusters and are not subjected to uniform control. Thus, their expression followed different time profiles with some ribosomal genes (e.g., *rpsF*, *rpsT*, *rpsU*, *rplD*, *rplM*, *rplU*, *rplY*) showing also strongly decreased expression after the onset of acetate uptake. All ribosomal genes nearly returned to their pre-glucose pulse expression level after acetate exhaustion. The increase of the expression of ribosomal genes after glucose addition demonstrates the cellular attempt to augment anabolic capacity. However, the biomass increase was only marginal in particular in the first phase during consumption of excessive glucose.

## Discussion

*Escherichia coli* adapted to carbon-limiting conditions is usually geared for energy-efficient carbon utilization. This includes also the efficient utilization of glucose, which serves as a source for cellular building blocks as well as energy. Thus, catabolic and anabolic functions are balanced under these conditions to minimize wasteful carbon utilization. Exposure to glucose excess interferes with the fine-tuned coupling of anabolism and catabolism leading to the so-called carbon overflow metabolism noticeable through acetate formation and eventually growth inhibition.

### Instantaneous response to glucose excess

The first obvious response of aerobically growing *E. coli* to sudden glucose excess or a glucose pulse is an instantaneous increase in respiratory activity (e.g., [15, 19, 20, 61, 62]; also this study). This instantaneous increase in oxygen uptake, noticeable even at high growth rates in carbon limited-fed batch cultures [63], suggests a great capacity to quickly expand respiratory metabolism. The respiratory burst is detectable within 10–20 s after glucose addition followed by a quick and usually sharp drop afterwards (e.g., [19, 62]; also this study). The following respiratory time course pattern as well as the extent of by-product formation depends on the amount of glucose added and other culture conditions. If the glucose pulse is low, by-product accumulation does not occur [61]. If the amount of glucose added is higher, by-product formation occurs but the initial respiratory response follows similar profiles: immediate sharp increase quickly followed by a drop in respiratory activity (e.g., [19, 20]; also this study).

Analysis of the energetic status of *E. coli* adapted to glucose-limited growth and subjected to suddenly increased glucose availability revealed in addition to the burst in respiratory activity a sudden increase in the ATP content as well as in the adenylate energy charge [20] pointing to a great potential to expand respiratory energy generation. However, after the initial burst phase in respiratory energy generation cells reduce respiratory activity concomitant to a decrease in the intracellular ATP content and adenylate energy charge to pre-glucose addition levels [20]. Thus, cells aim for energy homeostasis under conditions of glucose excess. It appears that cells are initially not prepared to utilize excess energy in anabolic pathways and thus reduce respiratory energy generation after the initial respiratory overshoot.

### Other (more delayed) changes of catabolism in response to glucose excess

The reduction of respiratory activity and continued uptake of glucose leads to accumulation of metabolites and forces carbon flow towards side reactions, e.g., to acetate, the most prominent overflow metabolite of *E. coli* but also to less energy efficient pathways such as the methyl-glyoxal pathway [58]. As the initial metabolic responses occur immediately after the glucose pulse they are certainly also regulated at the allosteric level of enzyme activity through accumulating metabolites auch as ATP, fructose-1,6-bisphosphate and pyruvate (please refer also to [20] for additional data and [64] for discussion). These initial responses towards glucose excess can reduce respiratory energy generation but they are not sufficient to lead to balanced catabolic and anabolic carbon processing. To further approach balanced metabolism it seems reasonable not only to change the metabolic enzyme activity but also the enzyme inventory. Again, changing concentrations of metabolites, e.g., cAMP and pyruvate are also involved in altering the transcriptome leading this way also to a change of the metabolic enzyme inventory.

Transcriptomic reorganization, mainly controlled through metabolites which modify TF activity, changes the metabolic enzyme repertoire, this way contributing to the adjustment of catabolic and anabolic cellular capacities. The initiated transcriptomic change is directed towards down-regulation of genes, which are required for active movement, carbon uptake and catabolic carbon processing, in particular to down-regulation of genes which contribute to efficient energy generation. Thus, cells change their enzymatic repertoire to reduce flux through the high-energy-yield pathways by enhancing the capacity for low-yield pathways in parallel as means to reduce ATP formation. This change includes also activation of those anaplerotic pathways which are connected to reduced ATP formation (e.g., enhanced expression of *ppc* instead of *pck*). In this line, previous experiments revealed that overexpression of NADH oxidases which decouple NADH oxidation from respiratory energy generation alleviated acetate formation while still keeping oxygen and sugar uptake high [65]. However, transfer of the transcriptome change into a modified enzyme repertoire takes time and is not sufficiently fast and effective enough to reduce catabolic carbon processing and prevent intracellular accumulation of potentially harmful metabolites in cells suddenly exposed to carbon excess. Thus, cells additionally enhance their capacity for exclusion of metabolites which they cannot utilize in anabolic pathways (e.g., formation and excretion of H_2_, acetate and glutamate, see also discussion section below).

Down-regulation of genes related to carbon catabolism occurs mainly at the level of substrate uptake and downstream of pyruvate and not in between in the glycolytic pathway. It is mainly accomplished through the reduced activity of CRP-cAMP and through an increased influence of phosphorylated ArcA. Former studies revealed that cAMP levels are usually high in chemostat cultures at steady state [66] suggesting an usually strong regulatory control through CRP-cAMP. These findings are in agreement with the here observed down-regulation of the CRP modulon, including the genes for motility and carbon uptake, upon the glucose upshift.

The general down-regulation of catabolic genes which are activated by CRP-cAMP as well as repressed by ArcA, can be partly compensated through the antagonizing interference with other TFs. For example, PdhR, which upon binding to pyruvate causes derepression of genes, can this way antagonize the expected reduction of the expression of pyruvate dehydrogenase genes (*aceE*, *aceF*, *lpd*) and some genes of the respiratory chain (*ndh*, *cyo*) (Fig. 5, Additional file 2, see also https://ecocyc.org/ [25, 47, 64]). Also, upon binding of pyruvate to the transcriptional repressor IclR, down-regulation of the genes of the glyoxylate bypass occurs (e.g., *aceA*, *aceB,* see also https://ecocyc.org/ [47]) as observed in response to the glucose pulse (Fig. 5, Additional files 1 and 2). Thus, metabolites are important to adjust catabolism, short term through allosteric regulation of enzyme activity and long-term through transcriptome changes leading to corresponding changes of the enzyme repertoire.

### Similar transcriptome changes occur in response to induced recombinant protein production

Similar observations were made in response to induced recombinant protein production which is often connected to growth inhibition and enhanced formation of metabolic by-products (e.g., acetate, pyruvate, glutamate [15, 67]). The transcriptomic response towards recombinant protein production was also mainly characterized by a vanishing influence of CRP-cAMP and an increasing influence of phosphorylated ArcA [25]. Energy-limiting conditions were also not detected, on the contrary, induced recombinant protein production was also initially accompanied by elevated ATP levels and an elevated adenylate energy charge [15, 64]. Recent experiments corroborated that interference of recombinant protein production with normal host cell metabolism is not the result of competition for energy and precursor metabolites but mainly an inhibitory effect of toxic amounts of recombinant mRNA on well organized and structured anabolic processes which are needed for cell growth [68–71]. The general cellular response towards recombinant protein production appeared to be in line with a response mimicking a carbon up-shift response required to match catabolic carbon processing with compromised anabolic capacities of induced cells [15, 25]. Thus, restrictions in anabolic pathways of different origins and ample or even excessive carbon supply can lead to similar responses.

### Other stress reactions and long-lasting changes in response to glucose excess

It has been proposed that cells exposed to carbon excess may induce the oxygen response regulon [59, 72]. We could not find any evidence for enhanced expression of genes related to the removal of reactive oxygen species. On the other hand, we could find evidence for a very strong and long-lasting induction of the acid response, especially the induction of the acid resistance system 2 (AR2). This appears at first puzzling, in particular as the cells did not face acidic conditions.

AR2, the so-called GABA shunt, is the most important acid resistance system which is induced at low pH and confers resistance to extreme acidic conditions [44]. Regulation of AR2 is very complex and not yet fully understood, e.g. expression control of *gadA* involves at least 13 TFs and the SFs RpoD and RpoS (see also https://ecocyc.org/) [47]. Moreover, metabolites such as pyruvate or metabolites down-stream of pyruvate are also thought to mediate acid resistance in *E. coli* [73]. The GABA shunt involves uptake of external glutamate, its decarboxylation to γ-amino butyric acid (GABA), a reaction which consumes a proton and the subsequent export of GABA towards the cells exterior [44]. Glutamate is not supplied in the medium employed in this study but it is known that glutamate is excreted into the culture medium in addition to pyruvate and acetate when carbon-limited cells are suddenly exposed to carbon (glucose) excess [15]. A closer investigation of the GABA shunt from a metabolic perspective reveals that in addition to protection against acidic conditions it can also contribute to reduced ATP formation and can be considered as part of an energy inefficient bypass of the TCA cycle. As in the TCA cycle α-ketoglutarate can be finally transformed into succinate utilizing part of the GABA shunt as TCA cycle bypass (see Additional file 1: Fig. S10). However, by utilization of the GABA shunt to bypass the direct step from α-ketoglutarate to succinate in the TCA cycle neither NAD(P)H nor ATP is being produced only glutamate being formed. Altogether this additional function of the GABA shunt can be interpreted as an additional means to reduce the formation of reducing equivalents and ATP as well as means to remove internal metabolites which cannot be used quickly enough in anabolic pathways.

It should be also noted that induction of the GABA shunt persists long after consumption of the surplus glucose and formed acetate potentially changing future fitness of carbon excess challenged cells. In this line, down-regulation of flagella-related genes also persists presumably also leading to long-term modification of cellular fitness.

Cells also aim to expand their anabolic capacities, e.g., through increased expression of ribosomal genes in response to the glucose pulse. However, increased expression of ribosomal genes did not translate into enhanced cell growth as the biomass increase was only marginal in particular in the first phase during consumption of excessive glucose.

Altogether, our analysis revealed that the major part of the metabolic response towards a glucose pulse is directed towards cell protection against excessive intracellular accumulation of metabolites including among others energy rich compounds such as ATP and extrusion of metabolites towards the cells exterior (e.g., acids, H_2_). Thus, resources are mainly utilized to cope with “overfeeding” and not for growth.

## Conclusions

Our findings suggest that cells are able to generate ample energy beyond their actual capacity for utilization in anabolic processes. If respiratory energy generation exceeds anabolic utilization an accumulating sensor metabolite or cell status (e.g., redox or energetic status) will lead on short-term to changes in metabolic fluxes through allosteric control of metabolic enzyme activities and long-term through modification of transcription factor activity to a change in the transcriptome and finally to reorganization of the metabolic enzyme repertoire.

The general change of metabolism in response to carbon excess is directed towards reduction of energy generation and catabolic carbon processing as well as towards protection against accumulating metabolites including reducing equivalents, acids and protons. Only a fraction of the metabolic change is dedicated towards expanded anabolic capacities.

Our findings shed light on carbon overflow metabolism resulting from exposure to sudden carbon excess and may even help to understand carbon overflow metabolism observed in balanced growth conditions such as carbon-limited growth at high growth rate where anabolic processes may also limit growth and not the catabolic supply of energy and precursor metabolites. Our findings are clearly not compatible with a general optimization principle aiming for energetic efficiency. It appears more reasonable to consider flexibility as well as an “unhealthy ravenous appetite” as principles leading to overflow metabolism. These principles might be operative in organisms which are naturally adapted to a carbon poor environment which they share with many different types of neighbors. Ample or even excessive carbon supply is not the typical challenge cells face in the intestine.

## Materials and methods

### Microorganism, medium and cultivation conditions

The *E. coli* K12 strain LJ110 (W3110 Fnr^+^) has been described before [74]. Cells were grown in glucose-limited continuous culture at 28 °C and pH 6.6 using a defined medium essentially as described previously (glucose concentration in the feeding solution 8 g L^−1^_,_ stirring rate 550 rpm, aeration 0.8 L min^−1^, VFerm 1 L) [12]. To mimic timely restricted feast conditions, a one-time glucose pulse was added directly to a steady state culture growing at a dilution rate of D = 0.072 h^−1^ suddenly increasing the glucose concentration to 10 g L^−1^. The concentrations of the other non-carbon nutrients (including nitrogen) were still above growth limiting concentrations. The continuous cultivation continued at otherwise unchanged conditions. The reproducibility in this experimental setup was verified in three biological replicates (Additional file 1: Fig. S1).

### Basic analytical techniques

Biomass was determined by measurement of the optical density at a wavelength of 420 nm. An OD420 = 1 corresponds to 0.18 gDCW L^−1^. Glucose was measured using the enzymatic test kit from Boehringer Mannheim/r-biopharm. Organic acids, i.e., acetic acid, lactic acid and formic acid were measured using the respective enzymatic test kits from r-biopharm. Pyruvic acid was analyzed enzymatically according to Bergmeyer [75]. The concentration of methylglyoxal was determined as described previously [76]. The concentrations of oxygen and carbon dioxide in the off-gas were measured by using the Magnos 14 and Uras 14 gas analyzer modules of ABB (Hartmann und Braun). Volumetric oxygen and carbon dioxide transfer rates (OTR and CTR, respectively) were calculated as described previously [12]. The dissolved oxygen concentration was analyzed using an inline probe for dissolved oxygen (Mettler-Toledo).

### Transcriptome analysis

DNA microarray hybridization and gene expression data analysis were carried out essentially as described previously [25]. Time-dependent transcript data were partitioned into clusters using a constrained k-means algorithm [77] with the background information of a combined transcription factor (TF)/sigma factor (SF)—target gene interaction from RegulonDB database (http://regulondb.ccg.unam.mx/) [21, 22]. From all time-dependent transcript data (4209 different transcripts in total, see Additional file 2) only those transcripts were considered for clustering which revealed at least for one sampling point an absolute log_2_ fold change of 1.5 after the glucose pulse (see Additional file 2). These differentially expressed genes (1739 DEGs in total) were sorted into clusters (see Additional file 2) with the optimum cluster number being determined by repeated calculation of the cluster validity index ‘Silhouette’ (see Additional file 1: Fig. S2) [78]. Following, a functional enrichment analysis (FEA) was carried out to determine which gene ontology (GO) categories are statistically over-represented in the clusters. In brief, the background information of Gene Ontology (GO) biological process (BP) from the whole genome as well as the DEGs were used for the FEA. Standard Fisher's exact test was used to calculate if a group of DEGs, which belongs to a certain GO BP category, was significantly enriched in a cluster. During the calculation using Fisher’s exact test: (1) the number of DEGs in a specific cluster, (2) the number of DEGs in this specific cluster which belong to a certain GO BP category, and (3) the number of genes in the whole genome which belong to this GO BP category, were used to calculate the corresponding nominal *p*-value of Fisher's exact test. All data are given in Additional file 2.

Finally, information from the RegulonDB database and the knowledge obtained through clustering was also used to generate a TF/SF target gene network matrix. Background information of TF/SF-target gene interaction from the whole genome as well as the DEGs were used for the generation of this matrix. Standard Fisher’s exact test was also used to calculate if a group of DEGs, which is controlled by a specific TF/SF combination, was significantly enriched in a cluster. During the calculation using Fisher’s exact test: (1) the number of DEGs in a specific cluster, (2) the number of DEGs in this specific cluster which are controlled by a specific TF/SF combination, and (3) the number of genes in the whole genome which are controlled by this TF/SF combination, were used to calculate the corresponding nominal *p*-value of Fisher’s exact test. All data are also given in Additional file 2.

To determine the most important over-represented GO categories or the most relevant TF/SF combinations, the following criteria were applied; number of differentially expressed genes with corrected *p*-values lower than 0.0025 belonging to a GO category or controlled by a specific TF/SF combination (either activated or inhibited) in one cluster is equal or larger than 9 (for details see Additional file 2). Correction of the raw *p*-values determined by Fisher’s exact test was done using the Holm-Bonferroni method [79]. The visualization of over-represented GO categories belonging to “biological process” [80] enriched in these clusters (see Additional file 2) was carried out using the WEB service “REVIGO” [23].

The knowledge generated by the TF/SF target gene network matrix, namely the identification of the most relevant TF/SF combinations governing the transcriptional response to the glucose pulse were used to analyze the time-dependent changes of the impact of single or multiple TF/SF combinations on the gene expression pattern. Control of gene expression through single or multiple TF/SF combinations was assessed through their sorting into different regulatory levels based on the number of TF/SF combinations controlling a set of genes. For example, 222 out of 4209 total genes activated by CRP-cAMP/RpoD and not co-controlled by the other identified important TF/SF combinations revealed reduced expression in response to the glucose pulse, thus the TF/SF combination (activation by) CRP-cAMP/RpoD is placed in Level 1. Or, as another example, 19 out of 4209 total genes which are activated by the combination of FlhDC/RpoD and not co-controlled by the other identified important TF/SF combinations exhibited reduced expression in response the glucose pulse, thus the TF/SF combination (activation by) FlhDC/RpoD is also placed in Level 1. Or, yet another example, 30 out of 4209 total genes activated by CRP-cAMP/RpoD and repressed by ArcA/RpoD but not controlled by any other of the identified important TF/SF combinations displayed reduced expression, thus the TF/SF combinations (activation by) CRP-cAMP/RpoD and (repression by) ArcA/RpoD are placed in Level 2 (for details see Additional file 2). From these important single or multiple TF/SF combinations those having a significant impact on the expression of controlled genes in response to the glucose pulse were determined by using Fisher’s exact test. Within each regulatory level correction of the raw *p*-values determined by Fisher’s exact test was done using the Holm-Bonferroni method [79] (for details see Additional file 2). To test whether the change of the expression of a set of genes controlled by a single or multiple TF/SF combinations at a given time point after the glucose pulse differs significantly from the change of other genes at this time point which are not controlled by this single or multiple TF/SF combinations, a one-sided two sample t-test was performed. Within each regulatory level the obtained raw *p*-values were again corrected using the Holm-Bonferroni method [79] (for details see Additional file 2). The impact of single or multiple TF/SF combinations on the change of expression of controlled genes in response to the glucose pulse was only regarded as significant if the corrected *p*-values obtained from Fisher’s exact test and t-test were both lower than 0.05.

## Supplementary Information

**Additional file 1**: **Fig. S1.** Metabolic response to the addition of a single glucose pulse to a glucose-limited continuous culture. Time course data of carbon dioxide transfer rates (CTR). The differently coloured curves correspond to three different independent experiments. Time zero indicates the time point of the glucose pulse. Experimental conditions as specified in the Materials and methods section of the main manuscript. **Fig. S2.** Cluster validity index ‘Silhouette’. Differentially expressed genes showing at least a log2 fold change of ± 1.5 in response to the glucose pulse were sorted into clusters using a constrained k-means algorithm [1] with the background information of transcription factor (TF)/ sigma factor (SF) - target gene interaction form RegulonDB database (http://regulondb.ccg.unam.mx/) [2, 3]. The optimal cluster number (N = 14) was determined by repeated calculation of the cluster validity index ‘Silhouette’ [4]. Mean and standard deviation of the cluster validity index ‘Silhouette’ are shown in the y-axis. **Fig. S3.** Detailed analysis of transcript changes in response to the glucose pulse from indicated pathways are given in Figs. S4 – S10. **Fig. S4.** Heat-map of time course profiles of transcripts (log2 fold change) encoding designated proteins involved in carbohydrate transport. **Fig. S5.** Heat-map of time course profiles of transcripts (log2 fold change) encoding designated proteins from pentose phosphate pathway, glycolysis and pyruvate decarboxy-lation. **Fig. S6.** Heat-map of time course profiles of transcripts (log2 fold change) encoding designated proteins from methylglyoxal metabolism. **Fig. S7.** Heat-map of time course profiles of transcripts (log2 fold change) encoding designated proteins from by-product metabolism. **Fig. S8.** Heat-map of time course profiles of transcripts (log2 fold change) encoding designated proteins from TCA cycle, glyoxylate shunt and anaplerotic reactions. **Fig. S9.** Heat-map of time course profiles of transcripts (log2 fold change) encoding designated proteins from respiratory energy generation. **Fig. S10.** Heat-map of time course profiles of transcripts (log2 fold change) encoding designated proteins from the GABA shunt (acid resistance system 2, AR2) and the mass, energy and redox balance of the GABA shunt (2-oxoglutarate to succinate). **Fig. S11.** Condensed heat-map of time course profiles of transcripts (log2 fold change) encoding designated proteins from central catabolic pathways.

**Additional file 2.** Transcriptome analysis. (Sheet 1) All time-dependent transcript data (4209 different transcripts in total, including regulation through single or multiple TF/SF combinations from those combinations identified later on as relevant). (Sheet 2) Time-dependent color-coded transcript data (heat maps) of genes responding differentially to the glucose pulse (1739 DEGs in total) sorted into respective clusters (14 clusters in total). (Sheet 3) Assignment to GO category “biological process” as well as the results of the functional enrichment analysis for determination of over-represented GO categories “biological process” (log10 nominal p-values of Fisher’s exact test). These data were used for the visualization of results presented in Fig. 2. (Sheet 4) 1739 DEG sorted into the 14 clusters together with their known control through all known TFs and SFs and the results of the functional enrichment analysis for determination of the most relevant TF/SF combinations (log10 nominal p-values of Fisher’s exact test). These data were used for the visualization of the results presented in Fig. 3. (Sheet 5) Selection of the most relevant TF/SF combinations. Time-dependent control of gene expression through single or multiple TF/SF combinations from those combinations identified as relevant. (Sheet 6) Fisher’s exact test (Sheet 7), corrected T-test and (Sheet 8) log10 T-test.

## Data Availability

All data generated or analyzed during this study are included in the published article and its additional files.

## Abbrevations

SF: Sigma factor
TCA: Tricarboxylic acid cycle
TF: Transcription factor
