# Supplementary material for: Is energy excess the initial trigger of carbon overflow metabolism? Transcriptional network response of carbon-limited Escherichia coli to transient carbon excess
Source: Microb Cell Fact. 2022 Apr 21;21:67. doi: 10.1186/s12934-022-01787-4 (PMC9027384; doi:10.1186/s12934-022-01787-4)
Supplement: Supplementary file 1 — Additional file 1: Fig. S1. Metabolic response to the addition of a single glucose pulse to a glucose-limited continuous culture. Time course data of carbon dioxide transfer rates (CTR). The differently coloured curves correspond to three different independent experiments. Time zero indicates the time point of the glucose pulse. Experimental conditions as specified in the Materials and methods section of the main manuscript. Fig. S2. Cluster validity index ‘Silhouette’. Differentially expressed genes showing at least a log2 fold change of ± 1.5 in response to the glucose pulse were sorted into clusters using a constrained k-means algorithm [1] with the background information of transcription factor (TF)/ sigma factor (SF) - target gene interaction form RegulonDB database (http://regulondb.ccg.unam.mx/) [2, 3]. The optimal cluster number (N = 14) was determined by repeated calculation of the cluster validity index ‘Silhouette’ [4]. Mean and standard deviation of the cluster validity index ‘Silhouette’ are shown in the y-axis. Fig. S3. Detailed analysis of transcript changes in response to the glucose pulse from indicated pathways are given in Figs. S4 – S10. Fig. S4. Heat-map of time course profiles of transcripts (log2 fold change) encoding designated proteins involved in carbohydrate transport. Fig. S5. Heat-map of time course profiles of transcripts (log2 fold change) encoding designated proteins from pentose phosphate pathway, glycolysis and pyruvate decarboxy-lation. Fig. S6. Heat-map of time course profiles of transcripts (log2 fold change) encoding designated proteins from methylglyoxal metabolism. Fig. S7. Heat-map of time course profiles of transcripts (log2 fold change) encoding designated proteins from by-product metabolism. Fig. S8. Heat-map of time course profiles of transcripts (log2 fold change) encoding designated proteins from TCA cycle, glyoxylate shunt and anaplerotic reactions. Fig. S9. Heat-map of time course profiles of transcripts (log2 fold ch [file 12934_2022_1787_MOESM1_ESM.pdf]

Additional file 1

Is energy excess the initial trigger of carbon overflow metabolism?

-Transcriptional network response of carbon-limited *Escherichia coli* to transient carbon excess-

Zhaopeng Li[1], Markus Nees[2], Katja Bettenbrock[2], Ursula Rinas[1,3]

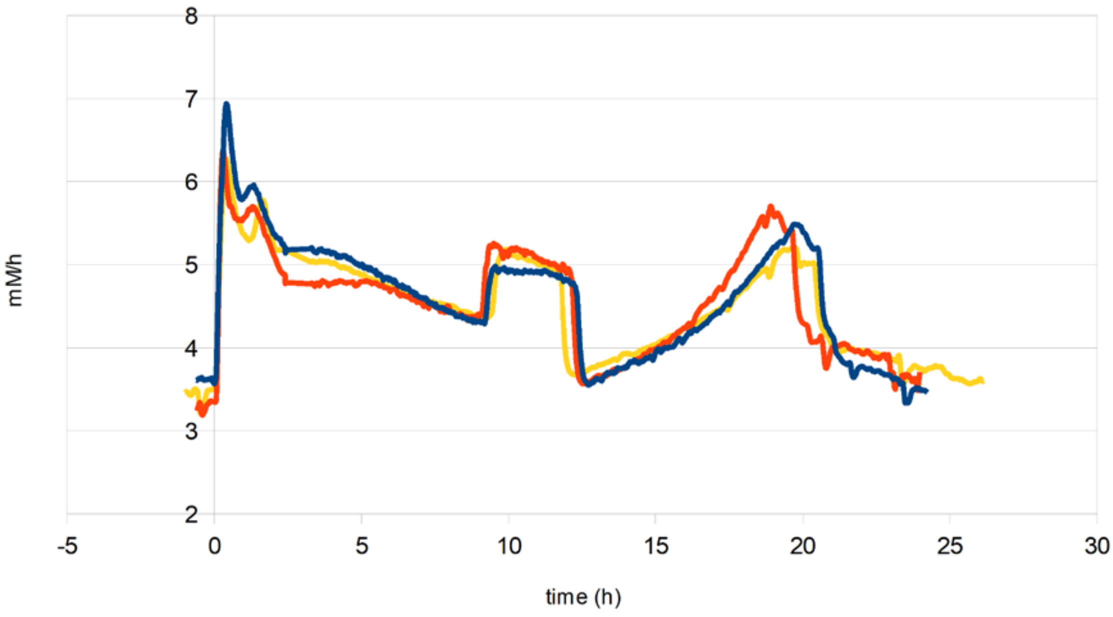

Fig. S1. Metabolic response to the addition of a single glucose pulse to a glucose-limited continuous culture. Time course data of carbon dioxide transfer rates (CTR). The differently coloured curves correspond to three different independent experiments. Time zero indicates the time point of the glucose pulse. Experimental conditions as specified in the Materials and methods section of the main manuscript.

Additional file 1

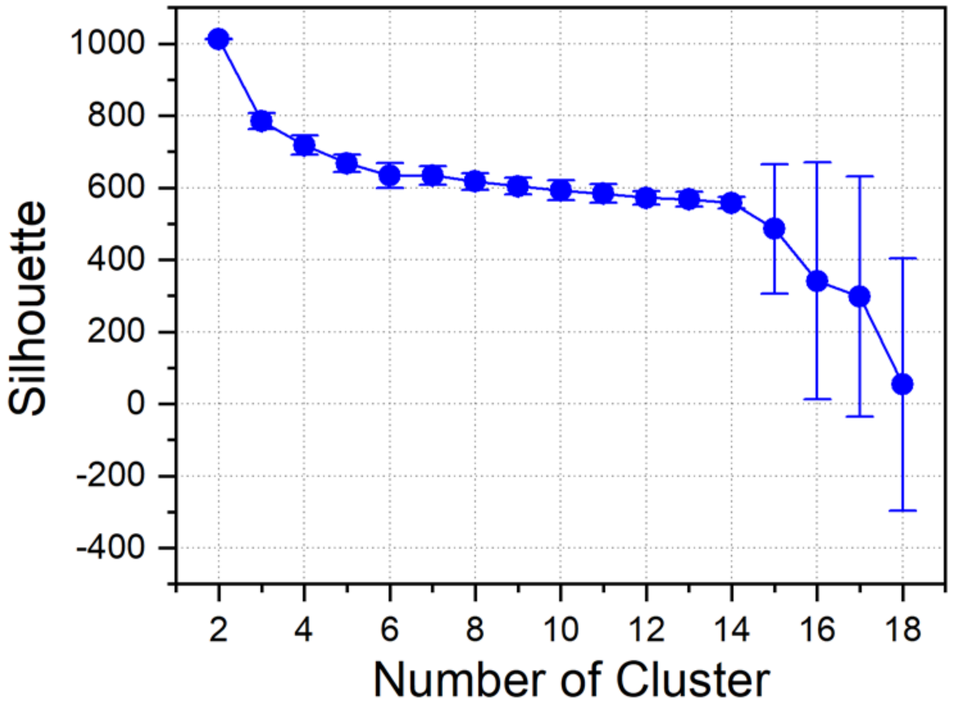

Fig. S2. Cluster validity index 'Silhouette'. Differentially expressed genes showing at least a $\log_2$ fold change of ± 1.5 in response to the glucose pulse were sorted into clusters using a constrained k-means algorithm [1] with the background information of transcription factor (TF)/ sigma factor (SF) - target gene interaction form RegulonDB database (http://regulondb.ccg.unam.mx/) [2, 3]. The optimal cluster number (N = 14) was determined by repeated calculation of the cluster validity index 'Silhouette' [4]. Mean and standard deviation of the cluster validity index 'Silhouette' are shown in the y-axis.

Additional file 1

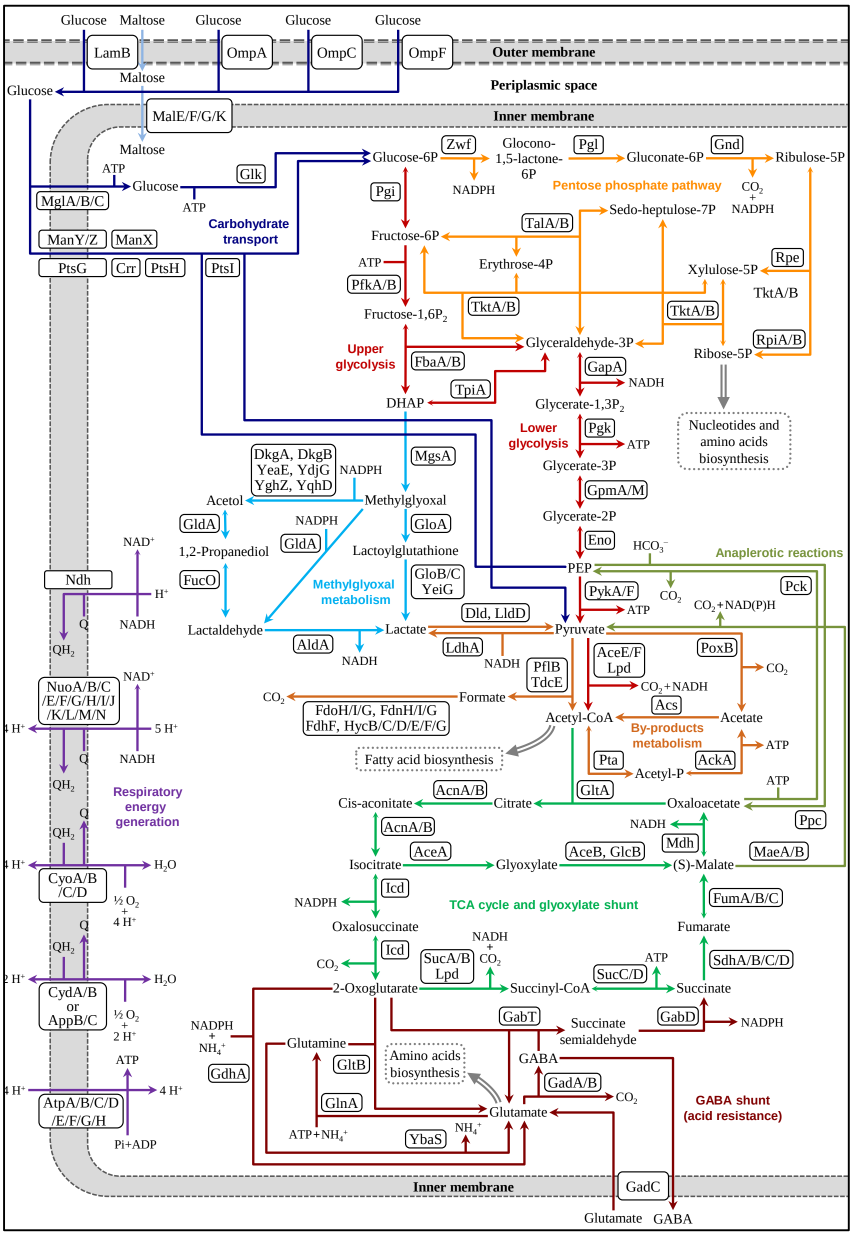

- Carbohydrate transport
- Pentose phosphate pathway
- Glycolysis and pyruvate decarboxylation
- Methylglyoxal metabolism
- By-products metabolism
- TCA cycle and glyoxylate shunt
- Anaplerotic reactions
- Respiratory energy generation
- GABA shunt (acid resistance)

Fig. S3. Detailed analysis of transcript changes in response to the glucose pulse from indicated pathways are given in Figs. S4 – S10.

Additional file 1

Gene	Log$_2$ ratio of transcription data																	
	-18 h	-0.5 h	0.5 h	2.5 h	4.5 h	6.5 h	8.5 h	9.5 h	10.5 h	11.5 h	13.5 h	15.5 h	17.5 h	19 h	20.5 h	24 h	25 h	26 h
**Outer membrane proteins**																		
ompA	-0.1	0.0	0.8	0.6	1.3	1.3	1.0	0.9	1.0	0.8	1.0	0.7	0.4	-0.5	0.2	-0.2	0.0	0.0
ompC	-0.2	0.0	0.3	0.3	1.3	1.8	1.9	1.9	2.0	2.2	2.1	2.1	2.0	1.2	1.3	1.4	1.5	1.4
ompF	-0.1	0.0	-1.0	-0.9	-0.9	-1.4	-1.9	-2.4	-2.4	-2.9	-2.7	-1.1	0.1	-0.2	-2.4	-1.6	-1.1	-1.4
lamB	0.3	0.0	-5.8	-8.4	-9.1	-9.6	-9.7	-9.6	-9.4	-9.8	-8.9	-7.9	-2.8	-0.3	-1.4	-0.8	-0.7	-0.7
**Transport of maltose (ABC transporter)**																		
malE	-0.2	0.0	-5.0	-8.4	-9.3	-9.6	-9.8	-9.8	-9.1	-9.6	-9.1	-7.1	-2.7	-0.3	-0.8	-0.8	-0.7	-0.6
malF	0.2	0.0	-5.8	-6.6	-6.8	-6.8	-6.8	-6.8	-6.3	-6.6	-5.9	-6.2	-3.3	-0.5	-1.3	-0.6	-0.9	-0.5
malG	0.4	0.0	-5.0	-5.7	-5.3	-5.3	-5.0	-4.9	-5.2	-5.0	-6.0	-5.1	-2.6	-0.1	-0.7	-0.2	-0.7	-0.2
malK	-0.6	0.0	-6.2	-7.6	-8.3	-8.6	-8.5	-8.5	-11.3	-8.5	-7.5	-5.4	-1.3	0.5	-1.0	-1.0	-0.8	-0.9
**Transport of Galactose/ Galactoside (ABC transporter, transport of glucose is possible)**																		
mglA	0.0	0.0	-5.1	-6.2	-5.8	-5.9	-5.9	-5.8	-5.0	-5.5	-5.5	-1.7	-0.8	0.7	-1.1	-2.0	-0.6	-1.5
mglB	-0.9	0.0	-5.8	-7.2	-6.4	-6.3	-6.1	-7.0	-3.7	-4.9	-2.9	-0.7	-0.4	0.7	-1.7	-0.9	-0.2	-0.6
mglC	-0.3	0.0	-4.8	-5.4	-5.3	-5.2	-5.2	-5.2	-4.7	-5.2	-4.9	-2.9	-1.4	0.6	-1.5	-1.1	-0.6	-0.9
**Transport of glucose by PTS systems**																		
manX	-0.2	0.0	-1.9	-2.5	-2.2	-2.0	-2.0	-1.9	-0.7	-0.8	-1.0	0.8	0.8	0.7	0.9	-0.1	0.2	0.1
manY	-0.1	0.0	-1.5	-2.2	-2.1	-1.8	-1.7	-1.7	-0.4	-0.6	-1.1	0.4	1.0	0.8	1.1	0.1	0.5	0.2
manZ	0.0	0.0	-2.0	-3.1	-2.2	-2.2	-2.2	-2.1	-0.6	-1.0	-1.0	0.0	0.5	0.3	1.3	0.5	0.4	0.3
ptsG	-0.5	0.0	-2.0	-0.9	-2.3	-2.2	-2.2	-2.3	-1.5	-1.9	-1.9	-1.0	-0.7	-0.7	-0.1	-0.3	0.1	-0.5
crr	-0.1	0.0	-0.2	-0.8	-0.2	-0.2	-0.3	-0.4	-0.3	-0.5	0.0	-0.6	-0.7	-0.8	0.1	0.3	0.3	0.3
ptsH	-0.1	0.0	-0.1	1.0	0.3	0.2	0.2	0.6	0.6	0.3	1.2	1.4	0.3	0.5	0.4	0.2	0.4	0.2
ptsI	0.0	0.0	-0.3	-0.1	-0.3	-0.2	-0.3	-0.3	-0.2	-0.3	-0.1	-0.3	-0.7	-0.6	0.2	0.3	0.3	0.2

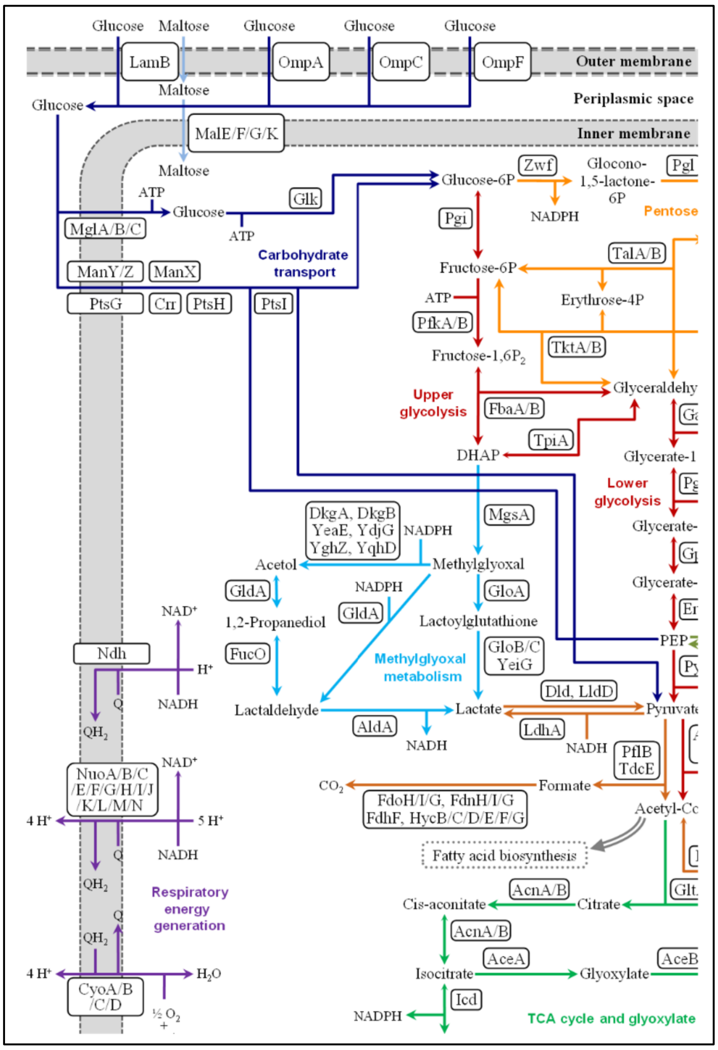

Fig. S4. Heat-map of time course profiles of transcripts (log$_2$ fold change) encoding designated proteins involved in carbohydrate transport. Time zero indicates the time point of the glucose pulse. Gene names are given to the left.

Additional file 1

Gene	-18 h	-0.5 h	0.5 h	2.5 h	4.5 h	6.5 h	8.5 h	9.5 h	10.5 h	11.5 h	13.5 h	15.5 h	17.5 h	19 h	20.5 h	24 h	25 h	26 h
	Log$_2$ ratio of transcription data																	
**Pentose phosphate phathway**																		
zwf	-0.1	0.0	0.9	0.5	0.7	0.9	1.0	0.7	0.7	0.7	0.8	0.5	0.0	0.1	0.5	0.3	0.4	0.3
pgl	-0.6	0.0	-0.4	-0.2	0.5	0.5	0.5	0.5	0.7	0.9	0.9	0.4	-0.2	-0.2	0.7	0.8	0.9	1.0
gnd	0.1	0.0	0.9	-0.1	1.7	1.7	1.5	1.4	1.0	1.0	1.8	1.0	1.4	0.9	0.2	0.1	0.4	0.3
tktA	-0.4	0.0	0.0	-0.7	0.1	0.0	0.3	0.1	-0.3	-0.2	-0.4	0.3	1.3	0.8	-1.2	-0.7	-0.2	-0.6
tktB	0.0	0.0	-0.4	0.2	0.5	0.6	0.6	0.6	0.6	0.6	0.5	-0.3	-1.2	-1.3	0.4	0.7	0.5	0.8
rpiA	-0.7	0.0	1.0	0.5	1.0	0.6	0.8	0.6	0.4	0.7	0.4	0.6	1.0	0.3	-0.1	-0.7	-0.3	-0.7
rpiB	-0.1	0.0	-1.5	-1.4	-1.7	-1.9	-1.7	-1.9	-1.7	-1.8	-0.8	-0.3	-0.4	0.3	-1.0	-0.8	-0.6	-0.7
talA	0.4	0.0	-0.3	0.0	0.6	0.5	0.5	0.5	0.6	0.7	0.8	-0.6	-1.7	-1.3	0.5	1.0	0.6	1.0
talB	-0.5	0.0	0.9	0.0	0.4	0.2	0.1	0.1	0.0	0.3	0.5	1.1	0.7	0.4	-0.7	-1.0	-0.3	-0.6
**Glycolysis**																		
pgi	-0.2	0.0	0.3	0.3	0.5	0.6	0.3	0.2	0.1	0.2	0.9	0.3	-0.1	-0.1	0.3	-0.2	0.2	-0.1
pfkA	0.2	0.0	0.4	1.8	0.7	0.5	0.3	0.5	0.0	0.1	-0.2	-0.2	-0.2	-0.2	0.7	0.3	0.3	0.2
pfkB	0.0	0.0	-0.2	0.3	1.1	1.3	1.8	2.0	1.9	2.2	-6.4	-6.3	1.6	1.4	2.3	2.0	1.6	1.8
fbaA	-0.1	0.0	0.2	0.4	0.6	0.7	0.8	0.9	0.6	0.6	1.1	0.6	0.7	0.4	0.3	0.4	0.7	0.6
fbaB	0.2	0.0	-0.9	-0.3	0.2	0.8	0.9	0.8	1.0	0.9	1.5	0.7	0.1	0.1	0.7	0.4	0.3	0.4
tpiA	0.1	0.0	0.7	1.4	0.9	0.9	0.9	0.9	0.9	0.7	0.6	0.9	0.6	0.5	1.2	0.4	0.7	0.4
gapA	-0.4	0.0	0.1	1.7	0.7	0.8	0.8	0.9	0.5	0.8	0.9	0.8	0.8	-0.1	-0.2	0.5	0.4	0.4
pgk	-0.2	0.0	0.6	1.4	0.8	0.5	0.3	0.4	0.4	0.4	0.5	0.3	0.0	-0.1	0.5	-0.2	0.1	-0.1
gpmA	-0.1	0.0	-0.7	-1.0	-0.1	0.1	0.1	0.0	-0.1	-0.2	-0.3	-0.4	-0.4	-0.7	0.0	0.3	0.3	0.1
gpmM	-0.1	0.0	1.5	4.0	1.6	1.9	1.8	2.3	1.1	1.2	0.9	1.3	1.1	0.7	0.7	0.5	0.5	0.5
eno	0.1	0.0	0.2	0.7	0.9	1.0	1.0	0.9	0.5	0.4	0.9	0.1	-0.2	-0.2	1.1	1.0	0.8	0.8
pykA	-0.4	0.0	-0.7	1.4	-0.1	-0.2	-0.2	-0.2	-0.2	-0.3	-0.5	-0.5	-0.2	0.2	0.2	-0.1	0.1	0.0
pykF	0.3	0.0	-0.5	0.0	0.2	0.5	0.7	0.7	0.5	0.3	-7.7	-8.1	-0.1	-0.3	1.2	1.4	1.1	1.2
**Pyruvate decarboxylation**																		
aceE	-0.1	0.0	2.0	0.7	0.5	0.3	0.2	0.3	0.3	0.4	0.5	-0.7	-1.1	-1.1	-1.4	-1.4	-0.8	-0.8
aceF	0.2	0.0	2.1	1.1	0.4	0.4	0.2	0.4	0.5	0.8	-0.1	-0.9	-1.3	-1.1	-1.8	-1.0	-1.0	-0.7
lpd	0.1	0.0	1.6	1.3	0.9	1.1	1.1	0.8	1.1	1.0	0.2	0.9	0.8	0.0	-0.9	-0.8	-0.5	-0.5

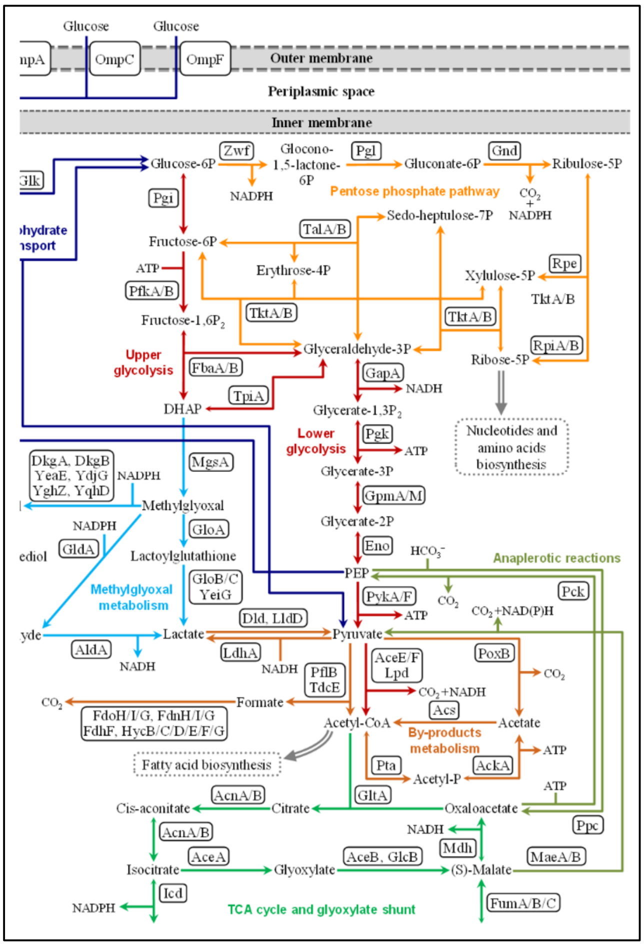

Fig. S5. Heat-map of time course profiles of transcripts (log$_2$ fold change) encoding designated proteins from pentose phosphate pathway, glycolysis and pyruvate decarboxylation. Time zero indicates the time point of the glucose pulse. Gene names are given to the left.

Additional file 1

Gene	Log$_2$ ratio of transcription data																	
	-18 h	-0.5 h	0.5 h	2.5 h	4.5 h	6.5 h	8.5 h	9.5 h	10.5 h	11.5 h	13.5 h	15.5 h	17.5 h	19 h	20.5 h	24 h	25 h	26 h
**Methylglyoxal metabolism**																		
mgsA	0.1	0.0	-0.7	-0.2	-0.8	-0.8	-1.0	-1.1	-1.0	-1.1	-1.3	-0.5	-0.4	0.0	-0.8	-0.7	-0.5	-0.6
gloA	-0.2	0.0	0.1	0.3	0.2	0.0	-0.2	-0.2	-0.3	-0.2	-0.1	-0.6	-0.6	-0.4	0.5	0.2	0.2	-0.1
gloB	0.9	0.0	0.1	0.3	-0.2	-0.1	-0.1	-0.1	-0.3	-0.4	-0.7	-1.1	-0.8	-0.1	0.9	0.8	0.5	0.6
gloC	0.0	0.0	1.0	0.9	1.1	1.1	0.8	0.8	0.6	0.6	0.1	0.6	0.5	0.2	-0.4	-0.3	-0.1	-0.3
yeiG	-0.2	0.0	0.2	-1.0	-0.3	-0.3	-0.3	-0.6	-0.4	-0.3	0.0	0.1	0.3	0.2	-0.9	-0.7	-0.7	-0.5
dkgA	0.7	0.0	-0.7	-0.4	-0.3	-0.2	-0.2	0.0	0.4	0.1	0.4	-0.1	-0.8	-0.1	1.3	1.5	1.0	1.5
dkgB	0.1	0.0	0.4	-0.7	-0.1	0.0	0.0	0.0	-0.2	-0.2	0.3	0.3	0.3	-0.2	-0.2	0.2	0.4	0.2
yeaE	-0.1	0.0	0.7	0.7	1.0	1.0	1.0	1.1	1.1	0.9	-7.1	-7.2	0.9	0.6	0.6	0.3	0.5	-0.2
ydjG	-0.7	0.0	0.4	0.4	0.5	0.4	0.3	0.3	0.0	-0.2	-0.7	-0.4	-0.1	0.4	0.3	0.0	0.2	0.1
yghZ	0.4	0.0	-3.4	-2.9	-2.8	-2.7	-2.7	-2.6	-1.6	-1.7	-1.1	0.0	0.2	0.7	0.4	0.4	0.1	0.4
yqhD	0.2	0.0	0.7	1.2	0.1	0.0	-0.1	-0.1	-0.2	-0.1	0.1	0.0	0.3	0.5	0.0	0.1	-0.1	0.4
gldA	-0.2	0.0	-0.1	3.7	-0.2	-0.1	-0.2	0.4	-0.2	-0.1	-5.9	-6.3	0.4	1.1	0.6	0.4	0.1	0.3
fucO	0.9	0.0	-1.7	-1.6	-1.6	-1.8	-1.7	-1.7	-0.9	-1.4	-0.6	0.1	0.8	1.3	0.5	-0.1	-0.1	0.4
aldA	-0.1	0.0	-6.8	-8.1	-7.9	-7.8	-7.8	-7.7	-3.3	-3.9	-3.4	-1.8	-1.2	-0.4	-0.7	-0.8	-0.1	-0.6

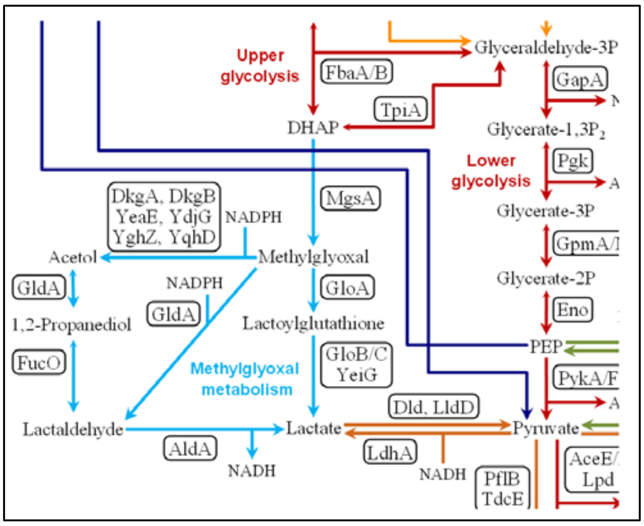

Fig. S6. Heat-map of time course profiles of transcripts (log$_2$ fold change) encoding designated proteins from methylglyoxal metabolism. Time zero indicates the time point of the glucose pulse. Gene names are given to the left.

Additional file 1

Gene	Log$_2$ ratio of transcription data																	
	-18 h	-0.5 h	0.5 h	2.5 h	4.5 h	6.5 h	8.5 h	9.5 h	10.5 h	11.5 h	13.5 h	15.5 h	17.5 h	19 h	20.5 h	24 h	25 h	26 h
**By-products metabolism**																		
**Acetate metabolism**																		
poxB	-0.2	0.0	-0.6	0.3	0.0	0.4	0.3	0.5	0.4	0.5	0.1	-0.2	-0.9	-0.9	0.2	0.3	0.2	0.4
ptA	0.3	0.0	0.7	2.3	0.2	0.5	0.5	0.6	0.4	0.3	0.3	0.3	-0.1	-0.1	0.3	0.5	0.4	0.4
ackA	0.2	0.0	1.0	2.6	0.7	0.3	0.2	0.8	0.3	0.2	-0.9	-0.8	-0.3	-0.2	0.6	0.4	0.4	0.2
acs	0.1	0.0	-6.3	-7.8	-6.8	-6.7	-6.7	-6.9	-4.3	-4.5	-3.3	-2.7	-1.9	-0.1	0.5	0.4	0.0	0.4
**Lactate metabolism**																		
dld	-0.3	0.0	-0.1	0.1	-0.1	0.1	0.0	-0.1	-0.3	-0.1	-0.4	-0.5	0.2	0.4	-0.7	-1.0	-0.6	-0.5
lldD	-0.5	0.0	-1.9	-1.3	-1.3	-0.9	-0.8	-0.9	-0.7	-0.7	-1.2	0.2	-0.1	0.5	-0.7	0.4	0.3	0.7
ldhA	0.2	0.0	1.3	1.6	0.8	0.8	0.7	0.8	0.9	0.8	0.8	-0.3	-1.2	-1.0	0.6	0.6	0.6	0.7
**Formate metabolism**																		
pflB	0.1	0.0	-0.1	1.5	0.3	0.5	0.5	0.5	0.3	0.4	0.5	-0.1	-0.1	-0.5	-0.2	0.4	0.3	0.5
tdcE	-0.1	0.0	0.7	1.4	0.8	0.8	0.8	0.8	0.6	0.7	0.0	-0.1	0.4	0.2	-0.5	-0.3	-0.2	-0.3
fdoG	0.3	0.0	-0.4	-1.1	-0.5	-0.5	-0.6	-0.5	-0.4	-0.6	-0.7	0.2	0.0	-0.2	-1.0	-0.1	-0.4	0.2
fdoI	0.3	0.0	-0.1	-0.2	-0.5	-0.7	-0.5	-0.4	-0.4	-0.4	-1.1	-0.4	-0.2	-0.2	-0.5	0.2	-0.2	0.3
fdoG	0.3	0.0	-0.4	-1.1	-0.5	-0.5	-0.6	-0.5	-0.4	-0.6	-0.7	0.2	0.0	-0.2	-1.0	-0.1	-0.4	0.2
fdnH	0.1	0.0	0.2	0.4	0.0	0.2	0.1	0.1	-0.3	0.2	0.7	0.4	-0.2	0.0	-0.2	0.0	0.1	0.0
fdnI	0.2	0.0	-0.2	-0.6	-0.2	0.0	0.5	0.3	0.1	-0.1	0.5	0.1	0.3	-0.1	-0.4	0.8	0.4	0.8
fdnG	0.0	0.0	0.1	1.0	0.8	0.4	0.2	0.2	-0.3	-0.3	0.0	2.8	0.2	0.0	0.8	0.9	0.2	-0.1
fdhF	-0.4	0.0	1.1	1.9	0.3	0.1	-0.1	0.1	-0.1	-0.1	0.8	-0.3	-0.4	-0.6	-0.3	0.0	-0.1	0.2
hycB	-0.3	0.0	1.5	6.3	0.8	0.6	1.0	1.7	1.2	0.8	1.8	0.2	-0.6	-0.3	0.7	0.3	-0.2	0.0
hycC	-0.7	0.0	1.1	4.3	0.7	0.8	0.8	0.4	0.5	0.8	1.4	-0.3	0.4	0.0	-1.3	-0.1	-0.1	1.0
hycD	0.4	0.0	0.7	3.0	0.5	0.5	0.5	0.4	1.3	0.2	1.1	-0.1	0.1	-0.1	-1.9	0.2	-0.2	-0.3
hycE	-0.9	0.0	0.6	0.6	0.5	0.5	0.6	0.6	-0.1	0.6	1.0	0.0	0.2	0.0	-1.9	-0.2	-0.5	-0.3
hycF	-0.1	0.0	0.0	0.1	0.2	0.0	0.0	0.0	0.1	0.0	0.9	0.1	-0.2	-0.1	-2.3	-0.5	-0.3	-0.3
hycG	-0.3	0.0	0.5	0.5	0.5	0.4	0.4	0.5	-0.2	0.3	0.7	1.1	0.2	0.0	0.3	-0.3	-0.2	-0.1

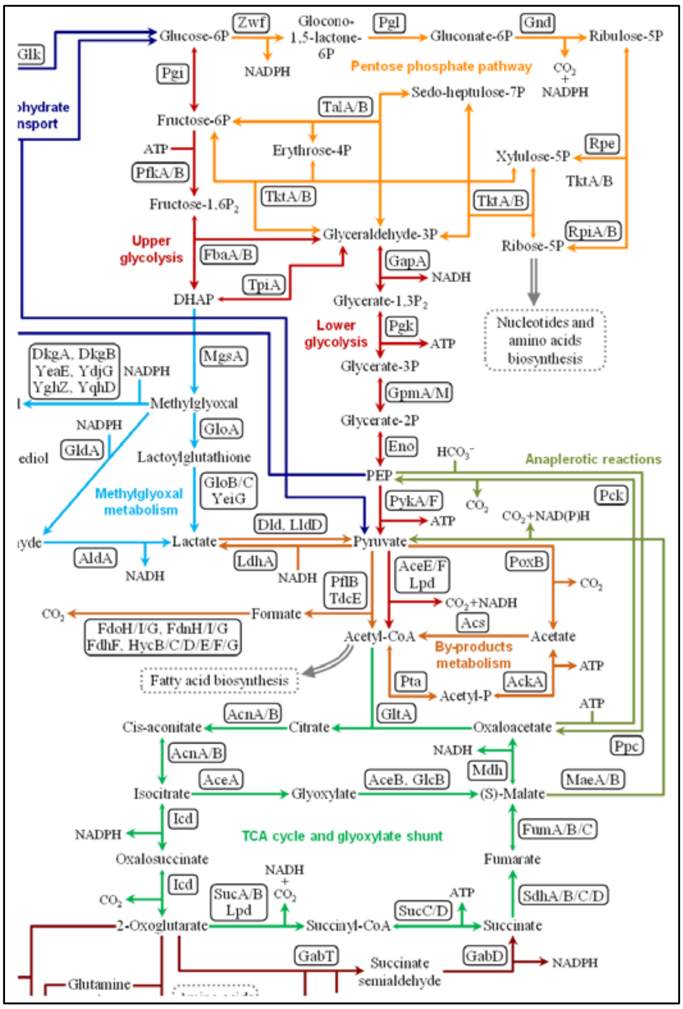

Fig. S7. Heat-map of time course profiles of transcripts (log$_2$ fold change) encoding designated proteins from by-product metabolism. Time zero indicates the time point of the glucose pulse. Gene names are given to the left.

Additional file 1

Gene	Log$_2$ ratio of transcription data																	
	-18 h	-0.5 h	0.5 h	2.5 h	4.5 h	6.5 h	8.5 h	9.5 h	10.5 h	11.5 h	13.5 h	15.5 h	17.5 h	19 h	20.5 h	24 h	25 h	26 h
**TCA cycle**																		
gltA	-0.2	0.0	-1.3	-3.7	-1.3	-0.9	-1.1	-1.2	-0.7	-0.7	-0.5	0.0	0.9	0.9	-1.7	-1.1	-0.6	-0.6
acnA	0.1	0.0	-1.0	-1.8	-0.6	-0.3	-0.5	-0.5	-0.2	-0.3	0.1	-0.5	-0.9	-0.4	0.7	0.6	0.2	0.6
acnB	-0.3	0.0	-1.3	-2.5	-0.6	-0.5	-0.6	-0.7	-0.8	-0.5	-0.4	-0.1	0.5	0.3	-2.1	-1.1	-1.0	-1.1
icd	-0.4	0.0	0.4	-0.4	0.5	0.4	0.4	0.2	0.3	0.3	0.0	0.5	0.8	0.4	-1.3	-1.3	-0.6	-1.1
sucA	-0.1	0.0	-3.1	-2.1	-2.3	-2.0	-2.0	-1.8	-0.4	-0.1	-0.7	1.3	1.4	1.1	-2.7	-1.4	-1.2	-0.9
sucB	0.0	0.0	-2.2	-1.7	-1.3	-1.5	-1.6	-1.4	-0.1	0.0	-0.2	0.8	1.5	1.1	-2.1	-1.3	-1.2	-0.7
lpd	0.1	0.0	1.6	1.3	0.9	1.1	1.1	0.8	1.1	1.0	0.2	0.9	0.8	0.0	-0.9	-0.8	-0.5	-0.5
sucC	-0.1	0.0	-1.9	-1.6	-1.2	-1.2	-1.4	-1.3	0.0	0.0	1.2	2.1	1.9	1.4	-2.0	-1.2	-1.0	-0.7
sucD	0.3	0.0	-2.3	-2.2	-1.6	-1.6	-1.7	-1.8	-0.2	-0.2	0.2	1.3	1.8	1.5	-1.7	-0.7	-0.8	-0.3
sdhA	-0.2	0.0	-4.1	-5.1	-2.4	-2.1	-2.3	-2.1	0.1	0.0	0.0	1.5	1.6	1.5	-2.3	-2.0	-1.3	-1.5
sdhB	-0.2	0.0	-4.0	-4.1	-2.4	-2.2	-2.2	-2.1	-0.3	-0.2	0.1	1.5	1.6	1.4	-2.5	-1.7	-1.2	-1.4
sdhC	-0.5	0.0	-3.7	-5.1	-2.0	-1.7	-1.4	-2.6	1.0	0.8	1.4	2.4	2.2	1.3	-2.0	-1.8	-1.1	-1.4
sdhD	-0.6	0.0	-3.7	-5.5	-1.9	-1.7	-1.5	-2.4	1.0	0.9	1.2	2.2	2.3	1.5	-1.8	-1.8	-1.1	-1.6
fumA	0.4	0.0	-1.9	-3.1	-1.1	-0.9	-0.9	-1.2	-1.1	-0.8	-0.3	0.6	1.5	1.5	0.7	0.5	0.5	0.7
fumB	0.0	0.0	-0.6	1.5	-0.3	-0.1	-0.3	-0.3	0.2	-0.4	-3.8	-4.0	0.9	0.6	-0.8	-0.7	-0.6	-0.5
fumC	-0.2	0.0	-1.8	-2.9	-1.9	-2.0	-2.3	-2.5	-2.4	-2.3	-2.2	-1.2	-0.7	-0.4	-1.6	-0.9	-0.8	-0.6
mdh	0.0	0.0	-0.8	-2.2	-0.3	-0.3	-0.1	-0.5	0.1	0.0	0.0	0.5	1.4	1.4	-0.1	0.0	0.1	0.2
**Glyoxylate shunt**																		
aceA	-0.2	0.0	-2.6	-3.2	-2.2	-2.2	-2.7	-2.3	-2.2	-2.1	-1.9	-1.6	-0.4	0.5	-0.3	0.1	0.0	0.0
aceB	-0.1	0.0	-1.9	-3.3	-1.3	-1.4	-1.5	-1.9	-1.5	-1.6	-1.3	-0.8	0.0	1.1	0.0	-0.4	0.6	0.1
glcB	0.2	0.0	-2.0	-1.8	-1.8	-1.8	-1.9	-2.0	-1.7	-1.1	-0.2	0.6	-0.4	0.2	0.0	0.1	-0.1	0.1
**Anaplerotic reactions**																		
maeA	-0.4	0.0	0.0	-0.2	0.0	0.1	0.1	-0.1	0.0	0.0	0.1	-0.2	0.4	0.3	-0.1	0.0	0.1	-0.2
maeB	-0.3	0.0	-0.5	-2.0	-0.9	-0.8	-1.1	-1.1	-1.0	-1.0	-4.2	-5.1	0.3	0.7	-0.6	-0.5	-0.4	-0.5
pck	-0.3	0.0	-2.6	-3.6	-3.6	-3.8	-4.0	-4.1	-3.3	-3.6	-4.6	-4.4	0.0	0.6	-1.0	-1.1	-1.2	-1.1
ppc	-0.5	0.0	0.8	0.7	1.0	1.3	1.1	1.1	0.5	0.7	0.2	0.3	0.3	-0.2	-0.9	-1.1	-0.5	-1.2

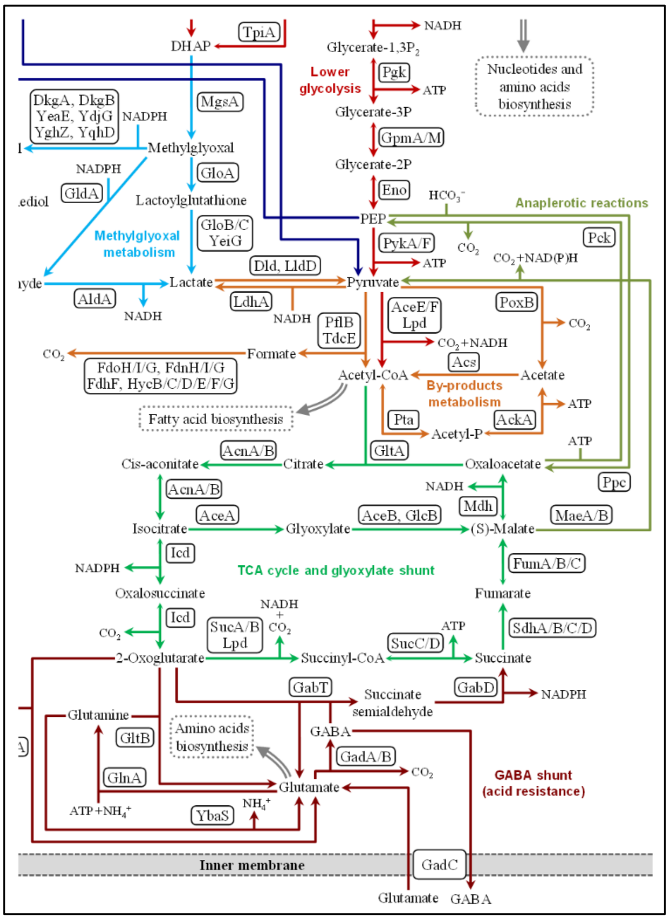

Fig. S8. Heat-map of time course profiles of transcripts (log$_2$ fold change) encoding designated proteins from TCA cycle, glyoxylate shunt and anaplerotic reactions. Time zero indicates the time point of the glucose pulse. Gene names are given to the left.

Additional file 1

Gene	Log$_2$ ratio of transcription data																
	-18 h	-0.5 h	0.5 h	2.5 h	4.5 h	6.5 h	8.5 h	9.5 h	10.5 h	11.5 h	13.5 h	15.5 h	17.5 h	19 h	20.5 h	24 h	25 h	26 h
**Respiratory energy generation**																		
**NADH dehydrogenase II (H^+/e^- = 0)**																		
ndh	-1.0	0.0	4.1	1.3	1.0	0.6	0.4	0.9	0.5	0.6	-0.4	-1.2	-1.6	-1.2	-0.8	-1.3	-0.4	-1.1
**NADH dehydrogenase I (H^+/e^- = 2)**																		
nuoA	-0.3	0.0	-1.1	-1.5	-0.7	-0.8	-0.8	-1.4	-0.7	-0.6	-0.7	-0.4	0.2	0.3	-0.4	-0.6	-0.4	-0.8
nuoB	-0.1	0.0	-0.8	-1.2	-0.2	-0.2	-0.5	-0.9	-0.6	-0.4	-0.3	-0.4	0.1	0.4	-0.3	-0.8	-0.4	-0.8
nuoC	-0.2	0.0	-1.3	-1.3	-0.7	-0.7	-1.0	-1.0	-0.9	-0.7	-1.1	-0.4	-0.2	0.4	-1.0	-0.9	-0.3	-0.7
nuoE	-0.1	0.0	-1.5	-0.9	-0.6	-0.7	-0.9	-0.9	-0.7	-0.6	-0.3	-0.1	-0.1	0.2	-0.6	-0.5	-0.5	-0.5
nuoF	-0.9	0.0	-1.3	-0.7	-0.5	-0.7	-1.0	-1.0	-1.1	-1.0	-0.9	-0.8	-0.4	0.3	-0.6	-0.3	-0.5	-0.2
nuoG	-0.1	0.0	-1.6	-0.7	-0.6	-0.7	-1.1	-1.2	-1.1	-1.0	-1.0	-1.5	-0.7	0.1	-1.0	-0.5	-0.5	-0.3
nuoH	0.2	0.0	-1.6	-0.5	-0.8	-0.8	-1.1	-1.1	-1.0	-1.1	-1.4	-1.0	-0.7	0.1	-0.9	-0.2	-0.1	0.1
nuoI	0.1	0.0	-1.8	-0.7	-0.8	-0.8	-1.1	-1.1	-1.1	-1.2	-0.8	-1.0	-0.7	-0.1	-0.7	-0.3	-0.3	-0.2
nuoJ	0.4	0.0	-1.5	-0.4	-0.5	-0.6	-0.9	-0.8	-0.8	-1.0	-0.7	-0.4	-0.2	0.2	-0.4	-0.1	-0.2	0.2
nuoK	0.3	0.0	-1.4	-0.3	-0.3	-0.4	-0.7	-0.6	-0.8	-0.8	-4.5	-6.5	-0.2	0.2	-0.4	0.0	-0.3	0.2
nuoL	0.0	0.0	-1.7	-0.6	-0.5	-0.6	-0.9	-0.9	-1.0	-1.0	-0.6	-0.8	-0.4	0.1	-0.8	-0.3	-0.2	-0.1
nuoM	0.1	0.0	-1.7	-0.4	-0.6	-0.5	-0.8	-0.8	-0.8	-0.7	-0.5	-0.4	-0.2	0.3	-0.5	-0.3	-0.3	-0.1
nuoN	0.0	0.0	-1.8	-0.6	-0.5	-0.5	-0.7	-0.8	-0.8	-0.7	-0.9	-0.8	-0.5	0.0	-0.7	-0.2	-0.3	0.0
**Cytochrome bo terminal oxidase (H^+/e^- = 2)**																		
cyoA	-0.3	0.0	1.2	-1.5	1.0	0.8	0.8	0.2	0.6	0.5	0.9	0.8	1.2	1.4	-0.5	-1.3	-0.3	-0.9
cyoB	-0.2	0.0	0.5	-0.7	0.6	0.4	0.1	0.2	0.0	0.2	0.3	0.4	0.6	1.0	-1.0	-1.0	-0.7	-0.6
cyoC	-0.1	0.0	0.7	-0.5	0.5	0.3	0.1	0.2	-0.3	-0.1	-0.2	0.2	0.5	0.9	-1.2	-0.7	-0.7	-0.6
cyoD	0.2	0.0	0.5	0.1	0.3	0.3	0.2	0.2	0.1	0.0	0.0	0.0	0.6	1.0	-1.1	-0.3	-0.4	-0.2
**Cytochrome bd-I and bd-II terminal oxidases (H^+/e^- = 1)**																		
cydA	-0.2	0.0	1.1	2.4	0.2	0.2	0.0	0.2	0.0	0.1	0.2	-0.2	0.2	0.5	0.3	-0.1	0.1	0.1
cydB	0.2	0.0	1.3	1.5	0.1	0.3	0.2	0.1	0.1	0.0	-0.3	-0.2	0.0	0.2	0.2	0.4	0.3	0.5
appB	0.0	0.0	0.2	0.6	0.9	1.4	1.5	1.7	1.8	0.6	2.2	1.8	1.1	0.8	0.9	0.8	0.4	0.7
appC	0.0	0.0	0.3	0.7	1.2	1.5	1.7	1.7	1.8	1.9	2.6	2.1	1.3	0.6	0.7	0.6	0.5	0.5
**ATP synthase (H^+/ATP = 4)**																		
atpA	0.2	0.0	-1.0	-0.5	-0.8	-0.9	-1.1	-1.0	-0.8	-1.0	-1.1	-0.1	0.5	0.7	-0.9	-0.8	-0.3	-0.4
atpB	-0.4	0.0	-0.6	0.1	-0.5	-0.8	-0.7	-0.8	-0.3	-0.6	-0.3	0.8	1.1	0.8	-0.4	-1.4	-0.1	-1.0
atpC	0.2	0.0	-1.1	-1.0	-1.4	-1.2	-1.3	-1.5	-1.6	-1.5	-0.8	-0.6	0.1	0.3	-1.3	-0.4	-0.3	-0.1
atpD	0.2	0.0	-1.1	-0.8	-1.0	-1.1	-1.2	-1.2	-1.1	-1.2	-1.1	-0.5	0.2	0.4	-1.0	-0.5	-0.4	-0.2
atpE	-0.1	0.0	-1.1	-0.5	-0.8	-1.1	-1.2	-1.3	-0.9	-1.1	-1.0	0.0	0.5	0.8	-0.4	-1.3	-0.5	-1.0
atpF	-0.3	0.0	-1.5	-0.8	-1.4	-1.8	-2.0	-1.9	-1.7	-1.9	-1.7	0.1	0.3	0.6	-1.0	-1.6	-0.6	-0.9
atpG	0.0	0.0	-1.5	-1.1	-1.3	-1.5	-1.6	-1.5	-1.6	-1.6	-1.3	-0.8	0.1	0.3	-1.2	-1.2	-0.6	-0.7
atpH	-0.2	0.0	-1.2	-0.6	-1.6	-1.3	-1.3	-1.2	-1.2	-1.3	-1.4	-0.2	0.3	0.4	-1.4	-1.3	-0.6	-0.8

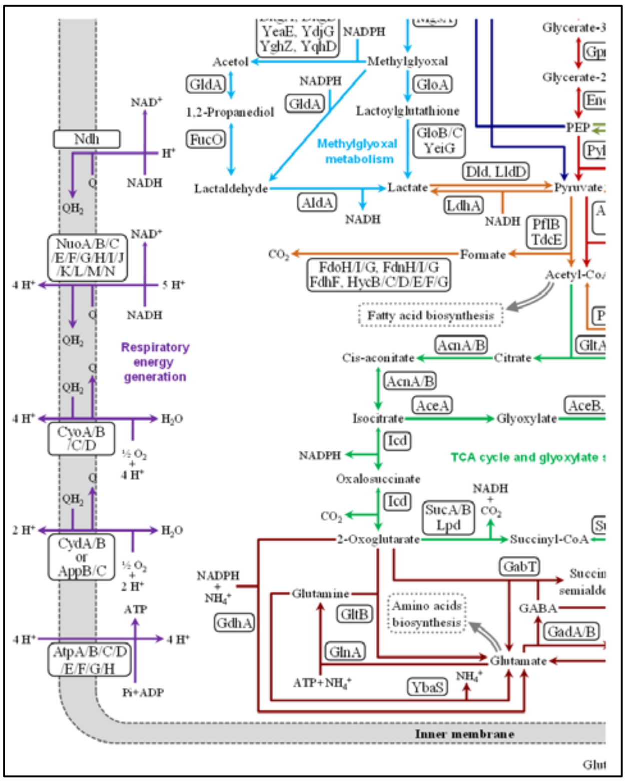

Fig. S9. Heat-map of time course profiles of transcripts (log_2 fold change) encoding designated proteins from respiratory energy generation. Time zero indicates the time point of the glucose pulse. Gene names are given to the left.

Additional file 1

Gene	Log$_2$ ratio of transcription data																	
	-18 h	-0.5 h	0.5 h	2.5 h	4.5 h	6.5 h	8.5 h	9.5 h	10.5 h	11.5 h	13.5 h	15.5 h	17.5 h	19 h	20.5 h	24 h	25 h	26 h
GABA shunt (acid resistance)																		
gabT	-0.3	0.0	-1.8	-1.1	-0.6	-0.1	-0.1	-0.1	0.3	0.5	0.9	0.3	-0.2	0.4	1.0	0.0	-0.2	0.0
gabD	0.0	0.0	-1.7	-1.3	-0.9	-0.4	-0.4	-0.4	-0.1	0.0	0.8	-0.4	-0.7	0.4	0.9	0.2	-0.1	0.2
gltB	0.1	0.0	0.9	0.2	0.5	0.5	0.3	0.3	-0.1	0.2	0.7	0.3	-0.3	-1.1	-2.2	-1.2	-1.1	-0.9
gdhA	-0.5	0.0	1.1	2.0	1.3	1.2	0.8	0.6	0.5	0.7	0.1	0.2	0.9	0.5	-0.9	-1.7	-0.7	-1.5
glnA	-0.7	0.0	2.2	2.8	1.6	1.5	1.8	2.6	1.2	1.2	1.1	1.5	2.6	1.3	-0.5	-1.2	-0.1	-0.6
gadA	0.4	0.0	-1.7	1.3	3.3	4.2	4.7	4.9	5.2	5.2	5.7	4.9	3.9	3.0	5.0	4.1	3.9	4.0
gadB	0.1	0.0	-1.6	0.8	2.7	3.7	4.2	4.2	4.4	4.6	4.7	4.1	3.2	2.5	4.3	3.7	3.5	3.5
gadC	0.6	0.0	-1.2	0.5	3.0	4.1	4.7	4.8	4.8	4.7	5.3	4.3	3.0	2.2	4.5	4.3	3.9	3.9
ybaS	0.5	0.0	-1.4	1.4	2.3	3.0	3.3	3.6	3.8	3.8	3.1	3.6	2.3	2.0	2.2	2.1	1.9	2.1

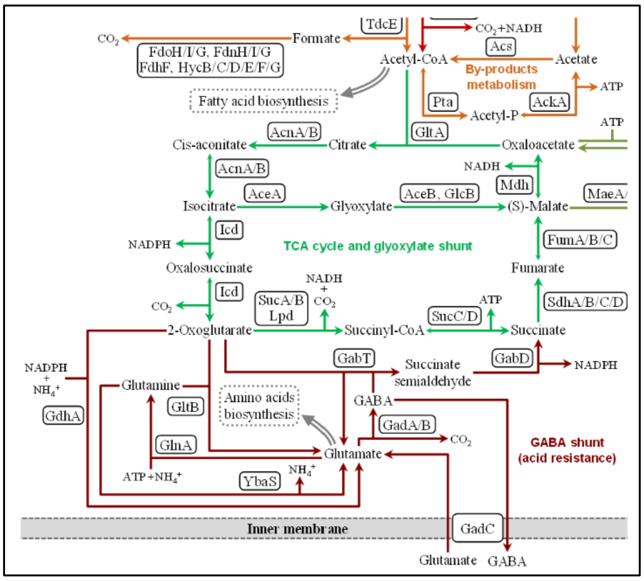

Fig. S10. Heat-map of time course profiles of transcripts (log$_2$ fold change) encoding designated proteins from the GABA shunt (acid resistance system 2, AR2). Time zero indicates the time point of the glucose pulse. Gene names are given to the left.

Mass, energy and redox balance: 2-Oxoglutarate to Succinate

1) **TCA cycle**

2 2-Oxoglutarate => 2 Succinate + 2 CO_2 + 2 ATP +2 NADPH

2) **TCA cycle bypass involving GABA shunt**

2-Oxoglutarate + NH_4^+ NADPH	=> Glutamate
Glutamate	=> GABA + CO_2
2-Oxoglutarate + GABA	=> Glutamate + Succinate-semialdehyde
Succinate-semialdehyde	=> Succinate + NADPH

Σ 2 2-Oxoglutarate + NH_4^+ => Succinate + Glutamate + CO_2

Additional file 1

Gene	Log2 ratio of transcription data																	
	-18 h	-0.5 h	0.5 h	2.5 h	4.5 h	6.5 h	8.5 h	9.5 h	10.5 h	11.5 h	13.5 h	15.5 h	17.5 h	19 h	20.5 h	24 h	25 h	26 h
**Methylglyoxal metabolism**																		
MgsA	0.1	0.0	-0.7	-0.2	-0.8	-0.8	-1.0	-1.1	-1.0	-1.1	-1.3	-0.5	-0.4	0.0	-0.8	-0.7	-0.5	-0.6
AldA	-0.1	0.0	-6.8	-8.1	-7.9	-7.8	-7.8	-7.7	-3.3	-3.9	-3.4	-1.8	-1.2	-0.4	-0.7	-0.8	-0.1	-0.6
lldD	-0.5	0.0	-1.9	-1.3	-1.3	-0.9	-0.8	-0.9	-0.7	-0.7	-1.2	0.2	-0.1	0.5	-0.7	0.4	0.3	0.7
**Pyruvate metabolism**																		
AceE	-0.1	0.0	2.0	0.7	0.5	0.3	0.2	0.3	0.3	0.4	0.5	-0.7	-1.1	-1.1	-1.4	-1.4	-0.8	-0.8
aceF	0.2	0.0	2.1	1.1	0.4	0.4	0.2	0.4	0.5	0.8	-0.1	-0.9	-1.3	-1.1	-1.8	-1.0	-1.0	-0.7
Lpd	0.1	0.0	1.6	1.3	0.9	1.1	1.1	0.8	1.1	1.0	0.2	0.9	0.8	0.0	-0.9	-0.8	-0.5	-0.5
**Acetate metabolism**																		
Acs	0.1	0.0	-6.3	-7.8	-6.8	-6.7	-6.7	-6.9	-4.3	-4.5	-3.3	-2.7	-1.9	-0.1	0.5	0.4	0.0	0.4
PtA	0.3	0.0	0.7	2.3	0.2	0.5	0.5	0.6	0.4	0.3	0.3	0.3	-0.1	-0.1	0.3	0.5	0.4	0.4
AckA	0.2	0.0	1.0	2.6	0.7	0.3	0.2	0.8	0.3	0.2	-0.9	-0.8	-0.3	-0.2	0.6	0.4	0.4	0.2
**TCA cycle**																		
GltA	-0.2	0.0	-1.3	-3.7	-1.3	-0.9	-1.1	-1.2	-0.7	-0.7	-0.5	0.0	0.9	0.9	-1.7	-1.1	-0.6	-0.6
AcnA	0.1	0.0	-1.0	-1.8	-0.6	-0.3	-0.5	-0.5	-0.2	-0.3	0.1	-0.5	-0.9	-0.4	0.7	0.6	0.2	0.6
AcnB	-0.3	0.0	-1.3	-2.5	-0.6	-0.5	-0.6	-0.7	-0.8	-0.5	-0.4	-0.1	0.5	0.3	-2.1	-1.1	-1.0	-1.1
Icd	-0.4	0.0	0.4	-0.4	0.5	0.4	0.4	0.2	0.3	0.3	0.0	0.5	0.8	0.4	-1.3	-1.3	-0.6	-1.1
SucA	-0.1	0.0	-3.1	-2.1	-2.3	-2.0	-2.0	-1.8	-0.4	-0.1	-0.7	1.3	1.4	1.1	-2.7	-1.4	-1.2	-0.9
SucB	0.0	0.0	-2.2	-1.7	-1.3	-1.5	-1.6	-1.4	-0.1	0.0	-0.2	0.8	1.5	1.1	-2.1	-1.3	-1.2	-0.7
SucC	-0.1	0.0	-1.9	-1.6	-1.2	-1.2	-1.4	-1.3	0.0	0.0	1.2	2.1	1.9	1.4	-2.0	-1.2	-1.0	-0.7
SucD	0.3	0.0	-2.3	-2.2	-1.6	-1.6	-1.7	-1.8	-0.2	-0.2	0.2	1.3	1.8	1.5	-1.7	-0.7	-0.8	-0.3
SdhA	-0.2	0.0	-4.1	-5.1	-2.4	-2.1	-2.3	-2.1	0.1	0.0	0.0	1.5	1.6	1.5	-2.3	-2.0	-1.3	-1.5
SdhB	-0.2	0.0	-4.0	-4.1	-2.4	-2.2	-2.2	-2.1	-0.3	-0.2	0.1	1.5	1.6	1.4	-2.5	-1.7	-1.2	-1.4
SdhC	-0.5	0.0	-3.7	-5.1	-2.0	-1.7	-1.4	-2.6	1.0	0.8	1.4	2.4	2.2	1.3	-2.0	-1.8	-1.1	-1.4
SdhD	-0.6	0.0	-3.7	-5.5	-1.9	-1.7	-1.5	-2.4	1.0	0.9	1.2	2.2	2.3	1.5	-1.8	-1.8	-1.1	-1.6
FumA	0.4	0.0	-1.9	-3.1	-1.1	-0.9	-0.9	-1.2	-1.1	-0.8	-0.3	0.6	1.5	1.5	0.7	0.5	0.5	0.7
FumB	0.0	0.0	-0.6	1.5	-0.3	-0.1	-0.3	-0.3	0.2	-0.4	-3.8	-4.0	0.9	0.6	-0.8	-0.7	-0.6	-0.5
FumC	-0.2	0.0	-1.8	-2.9	-1.9	-2.0	-2.3	-2.5	-2.4	-2.3	-2.2	-1.2	-0.7	-0.4	-1.6	-0.9	-0.8	-0.6
MdH	0.0	0.0	-0.8	-2.2	-0.3	-0.3	-0.1	-0.5	0.1	0.0	0.0	0.5	1.4	1.4	-0.1	0.0	0.1	0.2
**Glyoxylate shunt**																		
AceA	-0.2	0.0	-2.6	-3.2	-2.2	-2.2	-2.7	-2.3	-2.2	-2.1	-1.9	-1.6	-0.4	0.5	-0.3	0.1	0.0	0.0
AceB	-0.1	0.0	-1.9	-3.3	-1.3	-1.4	-1.5	-1.9	-1.5	-1.6	-1.3	-0.8	0.0	1.1	0.0	-0.4	0.6	0.1

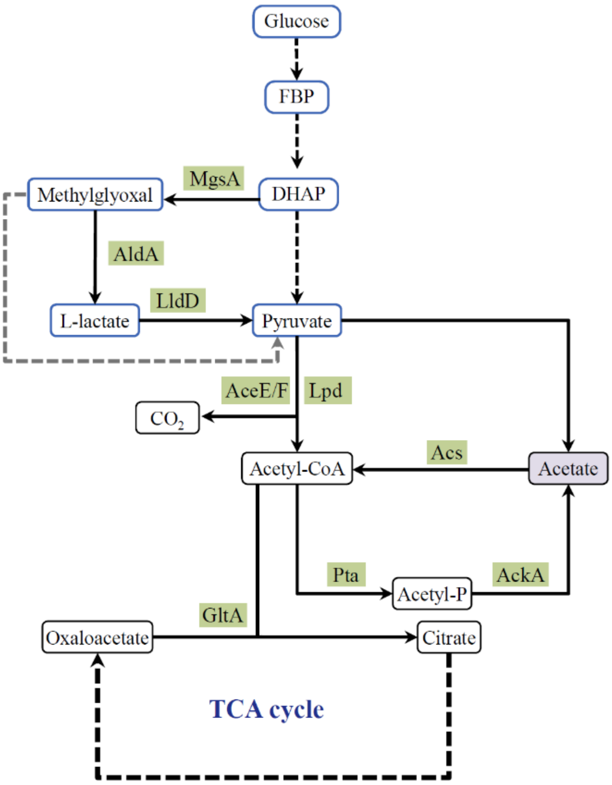

Fig. S11. Condensed heat-map of time course profiles of transcripts (log_2 fold change) encoding designated proteins from central catabolic pathways. Time zero indicates the time point of the glucose pulse. Gene names are given to the left.

Additional file 1

References

1. Wagstaff K, Cardie C, Rogers S, Schroedl S. Constrained k-means clustering with background knowledge. Proc. 18th Int. Conf. Machine Learning (ICML). 2001:577–84.
2. Gama-Castro S, Salgado H, Peralta-Gil M, Santos-Zavaleta A, Muniz-Rascado L, Solano-Lira H, et al. RegulonDB version 7.0: transcriptional regulation of *Escherichia coli* K-12 integrated within genetic sensory response units (Gensor Units). Nucleic Acids Res. 2011;39:D98-105.
3. Salgado H, Peralta-Gil M, Gama-Castro S, Santos-Zavaleta A, Muniz-Rascado L, Garcia-Sotelo JS, et al. RegulonDB v8.0: omics data sets, evolutionary conservation, regulatory phrases, cross-validated gold standards and more. Nucleic Acids Res. 2013;41:D203-D213.
4. Rousseeuw PJ. Silhouettes: a graphical aid to the interpretation and validation of cluster analysis. J. Comput. Appl. Math. 1987;20:53–65.
